# Systems biology of malaria explored with nonhuman primates

**DOI:** 10.1186/s12936-022-04199-2

**Published:** 2022-06-07

**Authors:** Mary R. Galinski

**Affiliations:** 1 Division of Infectious Diseases, Department of Medicine, Emory University School of Medicine, Atlanta, GA USA; 2 Emory Vaccine Center, Emory University, Atlanta, GA USA; 3 Emory National Primate Research Center (Yerkes National Primate Research Center), Emory University, Atlanta, GA USA

**Keywords:** *Plasmodium*, Host–pathogen interactions, Pathogenesis, Immunity, Systems immunology, Gametocytes, Rhesus macaques, *Macaca mulatta*, *Macaca fascicularis*, New World monkeys

## Abstract

“The Primate Malarias” book has been a uniquely important resource for multiple generations of scientists, since its debut in 1971, and remains pertinent to the present day. Indeed, nonhuman primates (NHPs) have been instrumental for major breakthroughs in basic and pre-clinical research on malaria for over 50 years. Research involving NHPs have provided critical insights and data that have been essential for malaria research on many parasite species, drugs, vaccines, pathogenesis, and transmission, leading to improved clinical care and advancing research goals for malaria control, elimination, and eradication. Whilst most malaria scientists over the decades have been studying *Plasmodium falciparum,* with NHP infections, in clinical studies with humans, or using in vitro culture or rodent model systems, others have been dedicated to advancing research on *Plasmodium vivax*, as well as on phylogenetically related simian species, including *Plasmodium cynomolgi*, *Plasmodium coatneyi*, and *Plasmodium knowlesi*. In-depth study of these four phylogenetically related species over the years has spawned the design of NHP longitudinal infection strategies for gathering information about ongoing infections, which can be related to human infections. These *Plasmodium*-NHP infection model systems are reviewed here, with emphasis on modern systems biological approaches to studying longitudinal infections, pathogenesis, immunity, and vaccines. Recent discoveries capitalizing on NHP longitudinal infections include an advanced understanding of chronic infections, relapses, anaemia, and immune memory. With quickly emerging new technological advances, more in-depth research and mechanistic discoveries can be anticipated on these and additional critical topics, including hypnozoite biology, antigenic variation, gametocyte transmission, bone marrow dysfunction, and loss of uninfected RBCs. New strategies and insights published by the Malaria Host–Pathogen Interaction Center (MaHPIC) are recapped here along with a vision that stresses the importance of educating future experts well trained in utilizing NHP infection model systems for the pursuit of innovative, effective interventions against malaria.

## Introduction

Malaria continues to be an intractable disease, ravishing communities in about 100 countries [1]. The cause of this disease—*Plasmodium* parasites transmitted by *Anopheles* mosquitoes—has been known since the late nineteenth century [2]. The World Health Organization’s Global Malaria Eradication Programme’s strategic use of the insecticide dichlorodiphenyltrichloroethane between 1959 and 1965 was halted, between costs concerns and as experts recognized that a multi-pronged approach would be required to achieve the goal of malaria eradication (reviewed in [3]). Once molecular biological methods took hold in the 1970s, attempts to make a malaria vaccine became the envisioned panacea (reviewed in [4]). These efforts continue today, with the full recognition that making and introducing an effective malaria vaccine(s) is no small task, especially since the ultimate protection worldwide will require vaccines that have long-lasting immunity and are effective against multiple species of *Plasmodium* with their ever-changing genetic variation (reviewed in [5–7]). Many factors must be considered to ensure the effectiveness of vaccines in malaria endemic communities [8]. Meanwhile, a panoply of rapid diagnostic tests has come to the forefront [9], complementing the traditional gold standard microscopy detection of parasites in blood smears, and drug development advancements continue, leading to new treatments and combination therapies to overcome rampant drug resistance [10]. Research has advanced incrementally, leading to an understanding of the life cycle of malaria parasites in their vertebrate and invertebrate hosts, the multiple species and strains of parasites and mosquitoes involved, as well as the pathogenesis, immune responses, and epidemiology attributed to each species in human populations. The rise of the Internet in the 1990s became critical in this long fight, enabling communication and progress among scientists and communities in ways not imagined in the past. Lately, the era of systems biology has emerged along with controlled human malarial infection (CHMI) studies, promising more in-depth understanding of malaria, host–parasite interactions and immunity.

This article highlights malaria research from early and recent understandings gained from malariotherapy and modern CHMI studies to NHP infection studies and their current utility in support of today’s malaria eradication goals, with emphasis on the value of longitudinal infections and systems biological approaches. This article also pays tribute to the authors of "The Primate Malarias” [11, 12], as this publication has been instrumental for scientists who have followed in their paths.

### Malaria parasite life cycles, within *Anopheles* mosquitoes and vertebrate hosts

**Fig. 1:**
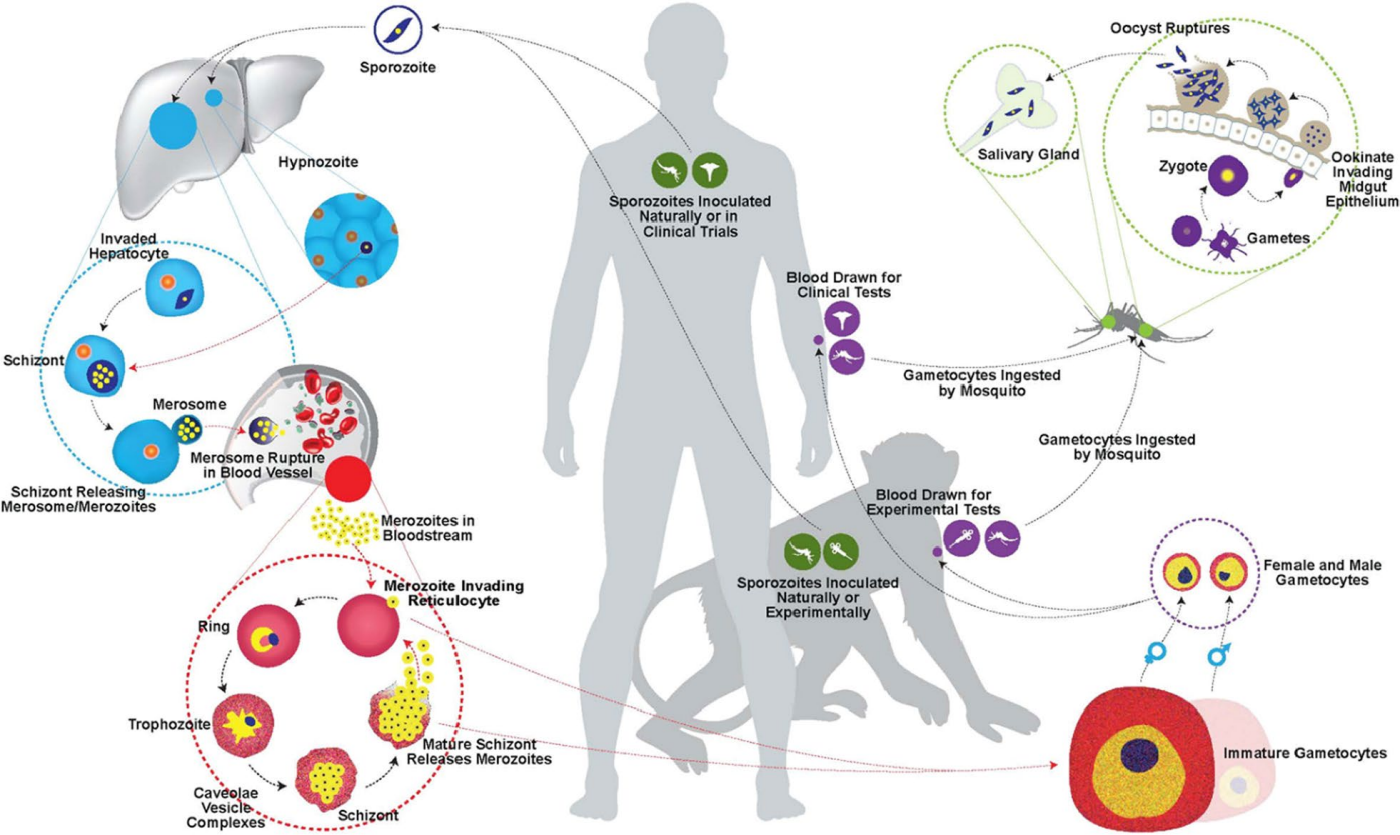
Schematic of the life cycle of *Plasmodium vivax* and comparable sibling simian species, depicted to represent the unique biological features of these species in the life cycle of primate malaria species and the importance of clinical and experimental interventions. The figure represents neotropical NHP models of *P. vivax* or macaque NHP models of *Plasmodium cynomolgi* and other simian parasite species that serve as surrogates for *P. vivax* (reviewed in [18–20])*.* The purple and green icons indicate where natural events and experimental manipulations can take place. The green mosquito icons refer to the natural inoculation of sporozoites through biting and the purple mosquito icons refer to the natural biting and infection of *Anophele*s ssp. mosquitoes by drawing in gametocyte-infected blood. The green medical symbol and syringe denoting the inoculation of sporozoites into the human and NHP hosts, respectively, refer to the possibility of challenging these hosts after immunization with a vaccine candidate to determine if protection can be induced. The purple medical symbol and syringe denote the collection of blood for testing involving human and NHPs, respectively. The purple syringe also signifies the specific collection of blood containing gametocytes from NHPs to artificially feed and infect *Anopheles* mosquitoes for supporting experiments on host–parasite biology within the vector host, transmission blocking vaccines, and access of sporozoites for in vivo or in vitro infection experiments. The unique biological features of *P. vivax* and comparable species depicted are the hypnozoite, the preferential invasion of merozoites into reticulocytes, the production of caveolae vesicle complexes (CVCs), represented as a mottled appearance of the infected RBCs, and the early and rapid development and circulation of gametocytes. Red arrows refer to processes relating to features that are currently in need of special research emphasis, answering questions like: (1) What is the make-up of hypnozoites and how are they activated? (2) What are the similarities and differences in primary and relapsing liver-stage schizonts and is their biology with merosome release in the blood stream comparable to rodent *Plasmodium* species where these were discovered? [21] (3) Which critical factors are required for reticulocyte host cell selection, invasion, and growth in these cells? And (4) what factors determine the development and circulation of gametocytes, potentially permitting transmission from the early stages of a blood-stage infection? “Reprinted from [19], with permission from Elsevier”. The artwork was created by Nagib Haque

Malaria parasites must successfully develop sequentially in the midgut and salivary glands of female *Anopheles* mosquito hosts and then be injected into the skin and survive in the liver and blood of humans, NHPs, or other vertebrate hosts. Some parasites must also transform within the blood of the vertebrate host into sexual-stage male and female gametocytes that are infectious for newly biting female *Anopheles* mosquitoes. The malaria parasite life cycles pose many challenges for both the parasites and hosts in their joint struggle to survive and propagate (Fig. 1). Throughout their transformative growth and development journey, the parasites must divide and multiply manifold, in the mosquito midgut, in hepatocytes, and inside red blood cells (RBCs). Importantly, throughout the process they must be able to overcome both innate and adaptive immune response barriers. Relapsing malaria parasite species, such as *Plasmodium vivax* (represented in Fig. 1, and discussed further below), have the added complexity of latent hypnozoite stages of the parasite in the liver and their possible activation that causes relapse parasitaemias in the blood, including the production of infectious gametocytes. Furthermore, *P. vivax* merozoites are restricted to young CD71+ reticulocyte host RBCs [13]. It is humbling to recognize the complexity of malaria parasites and their life cycles. Their size alone is large compared to viruses that are notoriously extremely difficult to control. For example, *Plasmodium* parasite genomes (discussed further below) comprise between 5000 and 7000 genes [14–16] versus 16 genes coded by the Severe Acute Respiratory Syndrome Coronavirus 2 (SARS-CoV-2) virus [17].

### Malariotherapy and controlled human malarial infections (﻿CHMIs)

From 1917 until 1976, malariotherapy was performed to treat patients with neurosyphilis and progressive paralysis (also known as general paralysis of the insane), as malarial infections caused fever, which can activate immune responses (reviewed in [22]). Over a period of 50 years (1923–1973), 17,000 patients were treated at the Mott Clinic for Malaria Therapy in England and numerous others underwent such therapy in Romania and the United States (reviewed in [23–26]). Aside from the great benefits to patients who recovered from severe neurosyphilis manifestations, malariotherapy provided the means to study *Plasmodium* infections longitudinally in humans, including the collection of parasitaemia datasets, thereby creating significant knowledge regarding clinical immunity and parasitological features of the disease, albeit in some cases with grave illness and deaths recorded as a result—from *Plasmodium falciparum*, *P. vivax,* and *Plasmodium malariae* infections. Up to 15 malarial paroxysms were observed and monitored in malariotherapy patients. Snounou and Pérignon provide an extraordinary overview of the literature from that time and the numerous retrospective analyses that have followed [23]. Their book chapter, titled “Malariotherapy—Insanity at the Service of Malariology,” is riveting. Indeed, the authors are correct to caution readers that the voluminous literature backing their chapter could become addictive! Malariotherapy was considered unnecessary once penicillin was discovered in the 1940s as a cure for syphilis. Subsequently, such human treatment measures—and the associated collection of experimental data—were deemed unethical and the practice was progressively stopped over the next 30 years. In the latter part of this period, many malaria research studies were instead carried out with prison inmates [27–30], and while ethical considerations were not addressed—to a similar extent as required today, the longitudinal infection data provided a basic understanding of malarial infections and disease progression. With gratitude shown for the participants, "The Primate Malarias” book is dedicated in part to the inmates at the United States Penitentiary in Atlanta, Georgia [28], who volunteered to accept infection with human and simian malaria parasites.

Many lessons were learned at the time, and since through the ongoing analysis of malariotherapy data (reviewed in [23]) and (also see [31–34]). These studies also ignited interest and establishment of mosquito insectaries for rearing and infecting *Anopheles* mosquitoes, from which enormous parasite-vector knowledge has been gained. Infection with *P. vivax* was most common. Retrospectively, many overarching conclusions still stand, and these can now be further explored taking into consideration the many since-discovered biological complexities of each parasite species, their *Anopheles* mosquito vectors, their epidemiology, ecological factors, strain diversity, parasite and host genetics, and the immune systems of either naïve or primed individuals, male or female, young and old (reviewed in [25, 35–38]). Some of the most prominent early discoveries beyond basic clinical and parasitaemic infection data include the demonstration of small exo-erythrocytic forms of *P. vivax* in liver-biopsies from a patient [39], now appreciated to likely have been hypnozoites (discussed below), the defining of the Duffy-blood group and its relationship with *P. vivax* susceptibility [40, 41], the dynamics of anaemia during *P. vivax* and *P. falciparum* infections and the conclusion that anaemia in acute cases is primarily caused by the removal of uninfected erythrocytes [31, 42–44], a process that has also been recognized as a “bystander effect” imposed on a majority of RBCs when a much smaller number of erythrocytes are actually infected (reviewed in [45]).

Today, CHMI studies involving volunteers infected with sporozoites have become a modern—and important—means to test anti-malarial drugs and vaccines and gather natural infection data from individuals over a series of experimental time points until blood-stage parasitaemia is detected and subjects require treatment (reviewed in [46–48]). In contrast to malariotherapy of the past, sporozoite-initiated CHMI studies have developed ethical standards that require treatment of individuals upon confirmation of parasites in the blood. Volunteers are monitored carefully and administered a full course of anti-malarial treatment that eliminates the parasites and the exacerbation of symptomology. As such, CHMI experiments have been very beneficial for studying *Plasmodium* infections from baseline periods to the time of parasitaemia, and helpful for conducting clinical vaccine trials that include sporozoite challenges (reviewed in [8]). Notably, correlates of protection are beginning to surface [49]. CHMI experiments initiated with sporozoites have also helped reveal specific protective immunological responses occurring during the early “pre-patent” period of infection, particularly in malaria-naïve hosts [50, 51], and new strategies for studying and intervening with gametocytaemia [52–56]. The time series data collected in CHMI studies are dynamic compared to cross-sectional data, traditionally generated for decades from patient samples in malaria endemic areas, and they have uncovered heterogeneous immune responses among individuals (reviewed in [57]). Additionally, of great benefit, piggybacked projects associated with CHMI experiments have provided insights into the epigenetic and gene expression characteristics of the resulting blood-stage parasites and their impact on immune cells [58–61].

CHMI studies have been mostly performed with *P. falciparum*, and close to a hundred such studies have been performed to date. Following suit, CHMI studies using *P. vivax* sporozoites have begun to emerge (reviewed in [62, 63]), including with vaccine trials [64–66]. CHMI research with *P. vivax* was initially stymied due to additional, unique challenges of attaining infectious *P. vivax* sporozoites [67–70] and the ability of hypnozoites to hide undetected in the liver and cause relapsing parasitaemias at some future point in time. Recent advances in the concentration of *P. vivax* gametocytes from infected volunteers [55] are helping to overcome the first of these hurdles, making it possible to generate *P. vivax* sporozoites from feeding *Anopheles* mosquitoes on well-characterized gametocyte-containing stocks and involving human volunteers with reliable infection records and medical histories. Thus, it may be possible in the future to perform such investigations routinely. At any rate, CHMI studies initiated with iRBCs are also now a viable option for investigations of *P. falciparum* [71–74], *P. vivax* [53, 55, 74–76] and *P. malariae* [77, 78]. Such CHMI studies involve low-dose blood-stage inoculations, with very clear ethical guidelines in place to ensure the safety of volunteers.

While CHMI experiments are generally safe and have demonstrated clear advantages for scientific advancements in understanding malaria, the viewpoints and concerns of participants and their communities and other stakeholders remain important to understand and take into consideration [79–82].

### NHP infection models (NHPIMs)

NHP infection models (NHPIMs) have been vital to research on malaria and over 70 human infectious diseases, including many caused by viruses, bacteria and other parasites [83, 84], and notably, recently for Ebola [85], Zika [86] and SARS-CoV-2 infection and coronavirus disease-2019 (Covid-19) [87]. With rapidly advancing technologies, NHP species chosen to best suit specific experimental questions and requirements increasingly hold much potential for complementary research on malaria, malarial immunity, and pathogenesis, as demonstrated in recent longitudinal experiments (discussed below). Given the close﻿ evolutionary relationship of humans and macaque species, many basic elements of the immune system are shared among them, making Old World monkey macaque species generally the most widely used NHP animal models for infectious disease research. Just as in humans, there is diversity in genes that comprise components of NHP immune systems. In turn, many reagents developed for the study of immune responses in mice or humans will not necessarily crossreact and be functional in studies of the NHP immune responses. However, various mouse, human, and NHP reagents have been confirmed to crossreact with conserved epitopes and others are under development and testing; e.g., see the NHP Reagent Resource web page [88].

Malaria with its myriad of host–parasite interactions throughout the parasite’s life cycle (Fig. 1), which are only beginning to be understood at the molecular level, is overwhelmingly complex. The complexity is compounded by the biological differences between species of *Plasmodium*, as elegantly detailed in “The Primate Malarias” [11, 12]. As also acknowledged by others [89], the authors of “The Primate Malarias” had the foresight to study and capture ‘now classic’ iRBC illustrations and basic morphological information regarding various human and NHP malaria parasite species and their life cycles in primates and *Anopheles* mosquito hosts, documenting nuances that distinguish species and strains, and experimental NHP and mosquito host infections. This all helped to set the stage for the use of different host–parasite combinations as model systems for research—whether for drug or vaccine candidate testing, immune response, or pathogenesis studies. The authors described infections of apes and Old World and New World monkeys, the most studied today being macaque (Old World) and *Saimiri* and *Aotus* (New World/neotropical) species. Numerous experiments had been performed to determine whether infections initiated by sporozoites of various species of *Plasmodium* infect and thrive in the liver and blood, or only the liver, and whether gametocytes were observed. Blood infections were studied including the level of infectivity of the NHP blood to mosquitoes. Numerous publications have followed since, by these authors and others, continuing to shine light on the utility of these model parasite species and the importance of NHP models for malaria research (reviewed in [18–20, 90–95]). The *Plasmodium*-NHP infection models have been and can continue to be useful for addressing specific hypotheses that may be challenging or not possible with humans, including when considering confounding factors among individuals within different populations and epidemiological and ecological situations worldwide (discussed in [25, 35, 96, 97]).

Differences in *Plasmodium* infections in splenectomized animals has also been documented in “The Primate Malarias” [11, 12] and is important to recognize. Humans and NHPs are more susceptible to higher parasitaemic blood-stage infections when lacking a spleen, and various experiments over time have been performed with parasites that have been ‘passaged’ through splenectomized animals and then frozen as iRBC stabilates for future use. From the 1960s until the turn of the twenty-first century, splenectomized chimpanzees had in fact been a source of occasional infectious samples to study the main four human malaria parasite species (*P. falciparum*, *P. vivax*, *P. malariae* and *P. ovale*) [98–102] (reviewed in [20]), but these types of experiments are no longer permitted, given ethical concerns and restrictions [103].

Importantly, while splenectomized NHPs have yielded higher parasitaemias of different species for various experimental purposes, and their use has been helpful to ensure the availability of long-term frozen infected RBC (iRBC) stocks for future use, such stocks and splenectomized animals are not advised for immunological and pathology studies seeking to understand the normal course of host–parasite interactions and disease ramifications. Aside from the spleen’s prominent role in removal of damaged RBCs, the spleen has been associated with the expression of parasite virulence factors, namely the *var/SICAvar* variant antigen genes and proteins at the surface of *Plasmodium*-iRBCs, specifically for *P. falciparum*, *P*.* coatneyi*, and *P*.* knowlesi,* and likely *P. fragile* (reviewed in [92]). *Plasmodium knowlesi*-iRBCs were shown to become less virulent after passage through splenectomized animals [104, 105], resulting in the downregulation of the expression of this gene family [106]. Splenic influence on antigenic variation has also been reported for *P. falciparum-*iRBCs in *Saimiri sciureus* monkeys [107] and in humans [108]. A study based on *Aotus* monkey infections has also indicated that the spleen influenced transcription of 67 *P. vivax* genes [109]. Moreover, the spleen has become recognized as a niche for a subpopulation of *P. vivax* iRBCs, possibly to promote their invasion and replication in host reticulocytes (reviewed in [110])*.* Interestingly in this regard, *P. cynomolgi* infections became attenuated when passaged in splenectomized rhesus [111]. The spleen may also be a niche for *P. cynomolgi.* While this remains to be explored, a computational model revealed that during infection of *Macaca mulatta* (rhesus monkeys), a subpopulation of *P. cynomolgi*-iRBCs becomes ‘concealed’ from the circulation [112]. In summary, the role of the spleen may prove to be distinctive for species that express *var/SICAvar* genes and proteins, compared to others that do not possess comparable genes and proteins. These distinctions are important as they relate to virulence and cytoadhesion and sequestration of iRBCs, or concealment of iRBCs, respectively.

Interestingly, the preface to “The Primate Malarias” noted that this book was timely in 1971 given the recent awareness that monkey malaria parasites can also infect humans [11, 12]. This comment well preceded the landmark studies of Singh et al. showing that human cases diagnosed in Malaysian Borneo as *P. malariae* were in fact *P. knowlesi* zoonoses [113]. Since that time, *P. knowlesi* has been documented as a zoonosis throughout most of Southeast Asia and as the main type of human-causing malarial disease in Malaysia [114]. More recently, *P. cynomolgi* has become recognized in the same geographical area as an additional zoonotic parasite of public health concern [115, 116] (reviewed in [89, 117]). Recently, adding to these concerns, a few cases of *P. coatneyi*, *P. inui, Plasmodium inui-like,* and *P. simiovale* malaria were identified in Malaysia when using molecular methods [118]. Geographical studies have been performed mapping *Macaca fascicularis* and the less-accessible macaque species *Macaca nemestrina* and *Macaca leonina,* highlighting their likelihood as well as possible hosts and sources of zoonotic *P. knowlesi* infections [119], and it is noteworthy that *Macaca arctoides* was recently identified as another macaque NHP species that can harbour this simian malaria parasite and be a possible source of zoonotic infections [120]. New World monkey infections and zoonoses involving *P. simium,* which is genetically and biologically similar to *P. vivax* [121], and *P. brasilianum*, which is genetically and biologically similar to *P. malariae* [89, 122], and *P. knowlesi* zoonotic infections are the subjects of other papers in this thematic issue.

Many basic biological and clinically relevant questions can be asked in NHP infection experiments (see Box 1). Longitudinal experiments can be designed without the urgent need to treat, and with repeat collection of multiple immunologically relevant tissues, thus capturing data not typically possible in CHMI studies. A holistic view of the immune response is possible with NHPIMs, especially with the Old World macaque models, including the collection and analysis of key tissues in addition to peripheral blood (e.g., skin, bone marrow, lymph nodes, liver, spleen) and integrated analyses of multiple data types during infections and from necropsies. Parenchymal tissues, secondary lymphoid tissues and the lymphatic/humoral circulation all hold relevant information. Over the past few decades, preclinical vaccine trials using NHP models have helped to test the immunogenicity of vaccine constructs, dosing, delivery platforms, and sometimes protection with parasite challenges. Such experiments have been cost-effective in the vaccine development pipeline. Today, these can be perfected to maximize these benefits.

**Table:** 

Box 1 Questions that can be addressed with NHP models
*Parasite biology*
• What molecular processes support hypnozoite development, maintain their dormancy, and trigger their activation? • How do hypnozoites manipulate hepatocytes to suppress innate immune mechanisms from inside the cell and preserve them until activation? • How do malaria parasites enter and remodel the infected RBC in vivo to suit their prime directive of multiplying and releasing new merozoite progeny? • What is the molecular genesis of gametocytogenesis of non-falciparum malaria parasites?
*Immunology*
• What are the mechanisms that thwart the formation of long-lived serological anti-parasite antibody responses, while memory cells remain persistent and protective? • What are the host–parasite mechanisms that enable antigenic variation and immune evasion? • What is the role of the spleen in these processes? • How does pre-existing immunity against malaria parasites impact vaccine-generated responses and subsequent infections with genetically heterologous parasites? • How do immune responses that are detected in the blood stream differ from what is occurring in various tissues and the systemic development of NAI and VCI? • What are key identifiable immune correlates of protection for NAI and VCI? • What immune responses and host molecules contribute to the development of anaemia?
*Pathogenesis*
• What systemic ramifications may occur due to the presence of LSFs in hepatocytes? • Can peripheral blood metabolites become diagnostic for hypnozoites? • What factors cause disease pathogenesis, inflammation, and pathology in various tissues? • What roles do extracellular vesicles play, or the microbiota, and in relation to immunity? • What factors contribute to loss of uninfected RBCs and bone marrow dysfunction? • What host-directed therapies can be developed to reverse adverse manifestations of malaria?
*Vector-parasite biology and transmission*
• The field is wide open for the application of the latest technologies to study mosquito infectivity, transmission, and subsequent host–parasite expression and interactions in NHPs

#### Old World monkeys—macaque species

Old World monkey species are valuable for researching the dynamic mechanistic intricacies of malarial naturally acquired and vaccine candidate-induced immunity (NAI and VCI), as well as gametocytogenesis and transmission. They also hold much potential for studying malarial pathogenesis and immune evasion mechanisms, and they are the preferred NHP species for longitudinal systems biological infection experiments. Significantly, the four known human malaria species (*P. falciparum*, *P. vivax*, *P. malariae* and *P. ovale*) do not develop in the blood of macaques. Simian malaria parasites, with comparable biology to the human malaria parasite species, are therefore relied upon when using these animal models. However, it is worth noting that while *P. falciparum* blood-stage parasitaemia does not develop in rhesus macaques, rhesus can be infected with *P. falciparum* up to the liver stage [123, 124]. Based on infection studies in *M. mulatta*, *P. coatneyi* has become recognized as a model for *P. falciparum*, *P. cynomolgi* as a model for *P. vivax*, and *P. knowlesi* as a model for both, depending on the research question. The history and prospects for using these species were reviewed recently for *P. coatneyi* [95], for *P. cynomolgi* [20], and for *P. knowlesi* in general terms [94] and with particular focus on antigenic variation [92].

The macaque NHP animal models develop a spectrum of malarial disease manifestations that closely resemble the human disease states caused by either human or simian malaria parasites. For example, *P. coatneyi* infections in rhesus macaques recapitulate malaria complications associated with pregnancy caused by *P. falciparum*, including with placental pathology and iRBCs localized in this tissue [125–128]. *Plasmodium coatneyi* infected rhesus also experience anaemia [129–131], with evidence of infected RBC sequestration and possible cerebral involvement [132–134]. Thus, this is a useful model for studying various aspects of *P. falciparum* pathogenesis (reviewed in [95]). In contrast, *P. cynomolgi* in rhesus macaques mirrors *P. vivax* relapse phenotypes [135–138], and this host–parasite pair has become recognized for studying primary and relapsing malaria parasite pathogenesis, with benefits understanding asexual and sexual stage immune responses and anaemia [139, 140]. Experimental studies with rhesus have also confirmed that *P. cynomolgi* causes placental pathology during pregnancy, which can result in severe pregnancy outcomes [141–144]. *Plasmodium knowlesi* infection of olive baboons (*Papio anubis*) has also been shown to be a model for studying malarial pathology including cerebral malaria [145] and malaria during pregnancy, with confirmed sequestration of iRBCs in the placenta [146]. Recently, Japanese macaques (*Macaca fuscata*) have been shown to be an additional viable NHP model for relapsing cynomolgi malaria [147]. Juvenile and adult macaques used for malaria research generally range from about 5–15 kg, allowing for adequate blood and bone marrow sample access at experimental time points and other tissues of interest selectively through biopsies or during necropsy.

Historically, malaria research involving macaque species has been carried out in the United States, the United Kingdom, The Netherlands, China, Thailand, India, Sri Lanka, and other parts of Southeast Asia. Research with these species is currently prominent in The Netherlands, Thailand, Japan, China, and the United States, where different species of macaques are routinely available through domestic breeding programmes or importation. In the United States, National Primate Research Centers (NPRCs), in addition to governmental laboratories, academic institutions, and private businesses, breed and/or may acquire macaque species for malaria research. Macaque species have been generally available as required for malaria research experiments, although at the time of this writing, their availability has been diminished due to their widespread use in SARS-CoV-2/Covid-19 experiments. This situation is likely to reverse, thanks to the development and testing of effective drugs and vaccines to fight the SARS-CoV-2/Covid-19 pandemic. Currently, as a result, severe Covid-19 illness has been subsiding [148].

#### New World monkeys—* Saimiri* and *Aotus* species

New World monkeys of the genus *Aotus* and *Saimiri* have been critical and will remain important for *P. falciparum* and *P. vivax* vaccine candidate trials, drug studies, and research into immune responses and pathogenesis caused by these parasite species, as well as basic research involving simian malaria parasite species (reviewed in [18–20, 26, 90, 91, 149, 150]). These monkey species have also been used to study the infectivity of the human parasite species *P. malariae* [102] and *P. ovale*, with the latter only shown to develop liver-stage parasites but not blood-stage parasites [101]. New World monkey systems-based research limitations include repeated tissue sampling and volume limitations imposed by their small size (~ 1 kg). In addition, specialized knowledge and husbandry are required to work with these animals, and there is currently a dearth of reactive biological reagents available for studying these species in systems-scale analyses [88]. Nevertheless, each of these models remain highly relevant to understand some aspects of immunity and pathogenesis. Data from each can improve upon interpretations of cross-sectional data from human studies and thus support intervention-based studies, and when ethical, support the collection of tissues to evaluate temporal changes that lead to specific clinical complications and can reveal hidden parasite niches (recent examples for *P. vivax* in *Saimiri boliviensis* and *Aotus lemurinus* include [151, 152]). Also, a recent insightful example of IgG, IgM, and IgA antibody isotype cross-reactivity relationships and lessons learned between *P. falciparum*-challenged human and *Aotus nancymaae* responses was shown using protein microarray technology [153–155]. Critically, as discussed below, the continued use of these animal models for generating infectious gametocytes for *P. vivax* research is paramount, particularly to research *P. vivax* hypnozoites and relapse biology. Historically valuable *P. vivax* isolates (over two dozen) that had been adapted to grow in New Word monkeys and stored as frozen iRBC stabilates are highly relevant today. These show individualized characteristics ranging from frequent early relapsing phenotypes (e.g., the Chesson strain) to late-relapsing phenotypes (North Korean strain) [18–20].

*Aotus* and *Saimiri* monkey species are currently available from the Keeling Center for Comparative Medicine and Research at The University of Texas MD Anderson Cancer Center, Texas, USA, which has maintained U.S. National Institute of Health (NIH)-supported New World monkey breeding colonies for medical research purposes, including malaria research. Opportunities for malaria research with New World monkey species also exist in Peru, Panama, Brazil, and Colombia.

## Twenty-first century—turning point in malaria research

Within this article, following an overview of relevant background information, systems biological studies and experimental frameworks are highlighted involving longitudinal NHP infections initiated with *P. coatneyi*, *P. cynomolgi*, and *P. knowlesi* sporozoites, from which basic biological knowledge has and continues to be gained regarding host infections initiated with each of these species. This research, conducted by the Malaria Host–Pathogen Interaction Center (MaHPIC), has involved longitudinal *Plasmodium* infections using macaque species, the most common being *M. mulatta* (rhesus monkeys) and *M. fascicularis* (kra monkeys, also known as long-tailed macaques, or cynomolgus monkeys).

### NHP research in the modern era of malaria systems biology and malaria eradication

It is well accepted that the host and parasite influence each other, and host–parasite interactions can set off cascades of molecular events and pathways, both locally and systemically, as each thrives to survive. The simian malaria parasites and their NHP model systems continue to provide unique research opportunities for in-depth investigations, aimed to ultimately inform translational solutions to prevent or treat malaria caused by any of the various *Plasmodium* parasite species infecting people. NHPIMs cannot recapitulate all health conditions and situations pertinent to humans, but they can begin to take them on, one or a few at a time; tease them apart, deconvolve large datasets, and reveal hidden biological information. It is becoming increasingly possible to gain a multi-dimensional view of the immune system in action, which is critical for determining: (a) what works to the host’s advantage, resulting in reduction or elimination of parasites, inflammation, and pathology, (b) what works to the host’s detriment, resulting in thriving parasites, illness, and pathology, and ultimately, and (c) what interventions may help favour the host. These three areas of inquiry remain at the forefront today.

### Malaria eradication goal raises the bar to tackle all species of *Plasmodium* and prioritize vaccines

In 2007, a new concerted effort to eradicate malaria began with a declaration by Bill and Melinda Gates to eradicate this disease [156]. Much debate followed [157] and continues today [158] on the feasibility of malaria eradication, and the challenges and bottlenecks faced [159, 160]. With the return of malaria eradication as a global goal, new projects were initiated to better document malaria cases and intervention methods being used regionally and globally. Gaps in knowledge were evaluated, and new research agendas developed to help fill those gaps, including for *P. vivax* which became recognized as a widespread but important and neglected species [161, 162]. These steps led to improved information about the prevalence, morbidity and mortality of both most predominant human malaria species, *P. falciparum* [163] and *P. vivax* [164, 165], as well as public recognition that global eradication will also require the elimination of the lesser prevalent human malaria species *P. malariae* and *P. ovale*, as well as simian malaria zoonotic species (reviewed in [89, 117, 118, 166, 167]). Three of these zoonotic species in particular, *P. cynomolgi, P. knowlesi*, and *P. coatneyi*, have been critical for studying malaria in vivo with NHP infections (reviewed in [92, 94, 95, 168]). Notwithstanding, *P. falciparum* research continues to predominate, given its well-known potential for serious illness and lethal outcomes, though *P. vivax* has gained recognition and research support as a parasite species that is widespread, debilitating, and which can be fatal (reviewed in [169]).

Researchers continue to question how to make the next big leap and meet the demands of malaria eradication [158, 160, 170, 171]. The successful evolution of host–parasite interactions favours the survival of both the parasite and its vertebrate and invertebrate hosts. The world’s challenge to break the cycle of malaria parasite transmission between—and development within—mosquitoes and humans is huge, yet dedicated researchers over the decades have remained committed to seeking sufficient understanding to interrupt transmission, foremost, with new vector control solutions, diagnostics, drugs, and vaccines [160]. Yet, much remains to be learned about the disease caused by the different *Plasmodium* species and their transmission dynamics, including ‘within-host’ and involving external factors, operating in a multitude of environments. Indeed, the enormous complexity of this overall system has led to new efforts utilizing mathematical and computational modelling strategies to study the problems and trajectories [172–174]. Top goals to achieve malaria eradication include treatment of infected individuals, whether they are symptomatic or not [175], with the goal of getting rid of all parasite subpopulations (circulating or not), including gametocytes [176] that transmit the disease to *Anopheles* mosquitoes where fertilization occurs and a new brood of parasites forms.

Vaccination is considered necessary to ultimately prevent infections and reduce (or eliminate) the global burden of malarial disease in humans (reviewed in [177]). While challenging to achieve, in-depth knowledge of malaria caused by each *Plasmodium* species, NAI, VCI, and malarial disease processes is critical for the development and testing of broadly effective vaccine strategies. Immunological comparisons between malaria naïve and chronically infected individuals are also important, particularly with regards to predicting and understanding the outcomes of candidate vaccine testing in malaria endemic areas with different transmission patterns and intensities, and when a large percentage of individuals may be repeatedly or chronically infected with malaria parasites and have significant levels of NAI against malaria.

### Raising the profile of *Plasmodium vivax* and its close relative, *Plasmodium cynomolgi*

Especially in light of the current malaria eradication agenda, *P. vivax* has gained widespread special attention as a predominant malaria parasite species—with raised public health concerns [161, 162, 178–184]—along with the closely related simian malaria species *P. cynomolgi* [185, 186]. Each of these species and a few others including *P. ovale* produce dormant parasite-infected cells in the liver that can stay quiescent for weeks, months or years and then become activated to cause relapsing infections in the blood in the absence of new mosquito infections. Consistent with raised attention on this problem, review articles on hypnozoites and relapses have become frequent [19, 20, 26, 187–194].

The biology of the *P. vivax, P. ovale *and* P. cynomolgi* latent parasites, called hypnozoites [195] (Fig. 1), is largely unknown. Hypnozoites comprise a large hidden subpopulation of the total *P. vivax* parasite biomass [196], and treatment options to kill them are currently limited to primaquine and tafenoquine, both with contraindications for pregnant individuals and anyone with glucose-6-phosphate-dehydrogenase deficiency; thus neither are ideal drugs [10, 166, 197]. Hypnozoites have low metabolic activity and are few in number in the liver, and therefore they have been a challenge to study and in turn discover essential biologically pathways to help advance non-haemolytic radical curative treatments. As such, they are a major barrier for malaria elimination and eradication campaigns [182, 184, 198–200]. The lack of robust in vitro parasite culture systems in the past has been considered an impediment to basic research on hypnozoites, but research with each of these species has remained viable over the past few decades thanks to the availability of ex vivo clinical samples from patients and NHP infections.

What causes these species to go dormant in hepatocytes—seemingly unaffected by the immune system; and what causes the dormant infected cells to reactivate and produce broods of thriving infectious parasites once again capable of causing blood-stage infections? While hypnozoites were discovered almost 40 years ago—in rhesus macaques (infected with *P. cynomolgi*) and chimpanzees (infected with *P. vivax*) [201–203], they were not researched with rigor until recently. Given recent investments and the persistence of scientists, hepatocyte culture systems have been established over the last several years for *P. vivax* [204, 205] and *P. cynomolgi* [206–208]. Robust *P. cynomolgi *in vitro blood-stage cultures [209] have also become a reality and these are enabling experiments that complement in vivo testing of hypotheses [210, 211]. Hepatocyte cultures are enabling hypnozoite transcriptomic data on *P. vivax* [204, 212–214] and *P. cynomolgi* [208, 215]. Recently, such in vitro cultures with *P. vivax* revealed parasite-derived membraneous networks in both schizonts and hypnozoites and possible functions of aquaporin-3 [216]. This type of research comes with many challenges, given the need to distinguish quiescent infected cells that have low metabolic activity from developing or newly activated forms [217, 218]. Today, such culture systems, single-cell -omic technologies [219], and cellular imaging advances [220, 221] are paving the way for using these model systems to understand *P. vivax* sporozoites [222] and host–parasite interactions in infected hepatocytes, including dormant and activated hypnozoites [223].

NHP models have become an important if not critical resource for enabling ongoing research with hepatocyte cultures. First, they have been vital for establishing gametocytaemia and feeding mosquitoes to generate *P. cynomolgi* and *P. vivax* sporozoites to initiate hepatocyte culture infections. Notably the first hepatocyte culture system to show results involved *M. fascicularis* and *P. cynomolgi*. *P. cynomolgi* blood-stage parasites were grown in macaques to generate gametocytes to feed mosquitoes to develop sporozoites to infect *M. fascicularis* hepatocyte cultures [207]. More in-depth studies can now be performed with NHPIMs, accessing *P. cynomolgi* or *P. vivax* gametocytes from infected NHP blood for mosquito feeds, and conducting ex vivo experiments with liver tissue samples.

Importantly, as the number of *P. vivax* clinical cases declines in endemic areas [1], the demand for NHP resources for investigating hypnozoites is expected to rise. Specifically, during the last decade, institutions in Thailand, Cambodia and India developed clinical capabilities and insectary operations to infect mosquitoes with human blood to attain sporozoites to infect hepatocyte culture systems [204, 205, 224]. These feats have been groundbreaking, but—if *P. vivax* clinical cases continue to decline, as hoped—logistical challenges will be compounded that include having sufficient availability of patient infected-blood donors, mosquito insectary operations, and experts for establishing, infecting, and analyzing the data coming from hepatocyte cultures. Meanwhile, there is much yet to be learned about the biology of hypnozoites to effectively identify targets for drug intervention, and multi-omic approaches that can distinguish host and parasite targets will be essential [218, 223]. With such knowledge and systems biological analyses, vaccination to eliminate hypnozoites, or at a minimum delay or reduce the number of relapses [225], may one day also become a reality, in addition to targeting the universal parasite developmental life cycle forms or stages known across all species as liver-stage forms (LSFs, or exoerythrocytic stages) and blood-stage forms (BSFs, or erythrocytic stages) (Fig. 1).

#### Relapsing malaria

Relapses are the ‘strong suit’ of *P. vivax* malaria and potentially *P. ovale*—for which less data is available [226–228]. *P. vivax* relapses are responsible for over 50% and potentially as high as 95% of blood-stage infections caused by these species [184, 198, 200, 229]. Yet, little to nothing is known specifically about these infections and immune responses generated against them. Malarial disease results from primary acute blood-stage infections with *Plasmodium* parasites, and recurrent infections. Studying relapsing malaria, caused by the activation of dormant *P. vivax* hypnozoites, is especially challenging, impractical—and arguably not possible—in human populations, where a main challenge is the difficulty of distinguishing relapse infections from new mosquito-borne infections. There are a few praise-worthy exceptions on the feasibility questions. Two major studies used molecular methods including whole genome sequencing and protein microarray analyses to distinguish new infections from relapses [230, 231]. Others involved the relocation of volunteers from malaria endemic areas of Cambodia to ‘non-transmission’ regions to ensure that any newly detected parasites in the blood were *bona fide* relapses [232, 233].

Fortunately, *P. vivax* relapses can also be studied via controlled experimental infections of New World monkeys, thus overcoming the challenge of discerning relapses from new infections in human studies. Monkey-adapted isolates of *P. vivax* can be capitalized upon that exhibit different relapse patterns (reviewed in [18–20, 26]). Aside from *P. vivax*, *P. cynomolgi* in macaques remains a strong model for in-depth investigations of relapses, driven by the fact that macaques are most closely related to humans and *P. cynomolgi* is a closely related sister species to *P. vivax* (reviewed in [20, 190])*.* Moreover, as noted above, *P. cynomolgi* is a zoonotic species in Southeast Asia and thus has direct relevance as an infectious disease agent for humans [115–117]. Critically, longitudinal studies of *P. cynomolgi* allow for controlled investigations that can distinguish one or more “*bona fide* relapses” from new infections or the rise or “recrudescence” of persisting low-level blood-stage parasites. Experimentally controlled relapses were first studied by Schmidt and colleagues using *P. cynomolgi* in rhesus macaques [135–137] and subsequently by others ([138, 139] and unpublished data) (see MaHPIC experiments below, called E04, E23, E24 and E25). Experimental infection of *M. mulatta* and other macaque species, initiated with *P. cynomolgi* sporozoites, can be designed to mimic primary, relapsing, and new infection occurrences, and such experiments are particularly well suited for studying host immune responses that develop during each of these distinctive blood-stage infections, including with parasite challenges involving homologous and heterologous strains of *P. cynomolgi*. As discussed below, systems biological approaches are being applied by the MaHPIC to assess the cascade of immune cell types, niches, and memory recall responses that result in naturally acquired protection against *P. cynomolgi*, while predictably enabling ongoing transmission to *Anopheles* mosquitoes ([139, 140] and unpublished data).

#### Gametocytes—the link to ongoing new infections

Circulating gametocytes remain undetected if an infected person is asymptomatic and not seeking treatment and there is no active case detection program in place in their community to identify and treat such individuals. This problem is especially challenging for primary infections with *P. vivax*, as the *P. vivax* gametocytes circulate soon during infection of the peripheral blood where they may be accessible to biting mosquitoes before individuals feel sick and seek medical attention [162, 235, 236]. Infectious gametocytes have also been confirmed in asymptomatic, chronic *P. falciparum, P. vivax,* and *P. malariae* infections, and they are, therefore, likely present as well in *P. ovale* infections [235, 237–242]. Further, they have been observed in *P. falciparum* CHMI studies soon after drug treatment [54, 243, 244]. Thus, for multiple and possibly all species, infectious gametocytes can be ingested by mosquitoes before anti-malarial drugs are taken or the immune system is effective, safeguarding their viability and ability to mate in the mosquito and propagate the disease. Today, longitudinal infections in NHPs provide opportunities to effectively study gametocytes and parasite transmission from a systems biological perspective under a variety of experimental conditions and scenarios [131, 139, 245, 246, 234].

#### Tissue niches: bone marrow and spleen

Cytoadhesion of *P. falciparum*-trophozoite and schizont-iRBCs to endothelial receptors and their concomitant sequestration in the deep vascular tissues have been known related phenomena for over 50 years, which lead to organ pathology, and explain why only the young *P. falciparum* ring-stage iRBCs are typically seen in peripheral blood smears (reviewed in [247, 248]). This is the case as well for the *P. coatneyi* and *P. fragile* simian malaria parasites with comparable ‘knobby’ iRBC surface structures and *var/SICAvar* genes and exposed adhesive variant proteins [249–254], predicted to be comparable to the adhesive *P. falciparum* Erythrocyte Membrane Protein-1 (PfEMP-1), which has domains known to adhere to various endothelial receptors [255, 256]. While cytoadhesion and sequestration have been proposed as evolutionary adaptations to avoid iRBC passage and destruction in the spleen, this is not fitting for the majority of *Plasmodium* species for which all developmental iRBC forms circulate. Questions remain regarding what other functions the adhesive PfEMP-1 and related proteins may serve for the parasites [257, 277].

Recently, an alternative concept called ‘concealment’ was put forth for species including *P. vivax* and *P. cynomolgi* to account for non-circulating iRBCs that do not express EMP-1 related proteins [24, 97, 112]. In these cases, other types of host–parasite interactions must account for the removal of subpopulations from circulation and possible associated disease manifestations. Above all, what has become clear, is that for all *Plasmodium* species, the circulating iRBCs represented in blood smear parasitaemias, do not necessarily represent the entire parasite load, or parasite burden in infected individuals (reviewed in [24, 97, 110, 196]). First, there can be multiple broods of parasites in an individual, each in different stages of the life cycle. Second, there can be an indeterminable number of parasites hidden in various tissues.

In the early stages of infection, *P. falciparum* gametocytes have been found in the bone marrow, where their maturation takes place [258–261], and the bone marrow has also been recognized as a development niche or reservoir for *P. vivax* gametocytes [262]. Surprisingly, the spleen has also become recognized as a biologically relevant tissue site for *P. vivax* iRBCs in humans [263–265], presumptively to support reticulocyte host cell invasion and the asexual blood-stage development cycle (reviewed in [110]). *Aotus* and *Saimiri* monkey NHP infection studies have demonstrated and quantified the burden of *P. vivax* iRBCs in these tissues relative to others [151, 152, 266]. Further studies using these animal models are now warranted, focused on local and systemic relationships of the infected bone marrow and spleen. Based on longitudinal parasitaemic data from *P. cynomolgi* infected *M. mulatta*, examined to better understand *P. vivax* infections and anaemia, a computational model was used and concluded that a subpopulation of iRBCs was in fact concealed in the tissues [112]. As with hypnozoites, these hidden parasite populations present bottlenecks for malaria eradication efforts.

*Plasmodium knowlesi* iRBCs have been identified in the tissues of experimentally infected *M. fascicularis* and *M. mulatta* [267, 268, 277]. In addition to vital lung, liver and kidney organs, among others, showing high levels of *P. knowlesi* parasitization, tissues of the gastrointestinal tract stood out, consistent with gastrointestinal complications in patients with *P. knowlesi* [268, 277]. *P. knowlesi* infection of baboons has also resulted in the localization of parasites in vital organs, including the brain and placenta [145, 146].

### Malaria vaccines, from darkness into the light

Throughout the 1980s, once a few malaria parasite genes had been characterized, a significant R & D focus developed towards a vaccine mimicking immunogenic regions of the parasite’s immunodominant circumsporozoite protein (CSP), hypothesizing that such a vaccine could prevent sporozoite infection of hepatocytes (reviewed in [4]). However, antibodies against this protein proved insufficient to inhibit infections completely, and once the parasites multiplied in even one liver cell, tens of thousands of invasive merozoites were subsequently released into the bloodstream. The need to raise effective B cell, T cell and other cell-mediated immune responses, and optimize adjuvants and delivery systems, took hold, resulting in vaccine renditions that included, among others, the CSP-based *P. falciparum* RTS,S candidate vaccine (reviewed in [4, 269–282]), and alternative CSP-based multi-valent vaccine plans for *P. vivax* [63, 65, 283, 284]. Recently, the *P. falciparum* RTS,S/AS01 vaccine product (Mosquirix™) was approved for widespread use in Africa after showing roughly 30% efficacy in preventing severe malaria in children after four sequential injections [285, 286]. This malaria vaccine will be the first to be widely implemented, and thus, it will allow the research community to observe, for the first time, the possible benefits as well as effects on local parasite genetics and dynamics, disease prevalence, and transmission that can result from the introduction of a subunit malaria vaccine based on an immunodominant protein representing a single species and strain [287, 288]. This achievement has and will continue to pave the way for testing of other currently available and future-generation vaccine candidates [280, 281, 289–293].

As it stands, the world is still far from being close to having one or more malaria vaccines that can completely prevent malaria from one species, let alone all four human *Plasmodium* species and zoonotic infections. Uncounted lessons have been learned during the roughly 30 years of RTS,S development, testing, delivery/administration, and monitoring phases. While sterilizing immunity—killing all parasites—has yet to be reached in large clinical trials, it must remain the goal as malaria vaccinology work continues alongside research to understand each parasite species’ biology, diversity, antigenic variation, and evasion strategies, as well as host immune responses, including those that are protective and those that may be detrimental and cause pathology. Moreover, differences in responsiveness between malaria naïve volunteers and people living in different malaria endemic areas must be considered (reviewed in [8, 294]). Ultimately, it is hoped that useful predictive immune signatures and correlates of protection will be identified, and that future vaccine candidates will be tested with much more relevant knowledge known about the likely, and requisite, host responses necessary to achieve protection, if not sterility (i.e., the complete elimination of parasites in an individual).

Since the 1990s, multipronged approaches have been increasingly pursued against multiple parasite targets and life cycle developmental stages, including the sexual stages (Fig. 1), in an attempt to block successful transmission and development of parasites within malaria endemic mosquito populations [295, 296]. Major investments over many decades have advanced malaria vaccine development as a top priority, among government agencies, industries, and foundations, including the Bill and Melinda Gates Foundation (BMGF). The European Malaria Vaccine Initiative (EMVI), launched in 1998, complemented several European malaria vaccine research initiatives [297]. The US-based Malaria Vaccine Initiative (MVI) was launched in 1999 with the benefit of $50 million in start-up support from the then newly developing BMGF, and the MVI leadership team scouted out and supported projects and approaches around the world that they deemed promising, mostly towards *P. falciparum* vaccines [298], but also including the *P. vivax* Duffy Binding Protein (PvDBP) as a one-shot key prospect immunogen to prevent entry of *P. vivax* merozoites into Duffy glycoprotein-positive host RBCs (reviewed in [299–301]). These DBP vaccine efforts follow a body of research showing the importance of the Duffy glycoprotein on the surface of RBCs [41] as a receptor for the *P. vivax* DBP, which is located at the front (apical) end of the merozoite and is known to specifically attach to the cognate RBC receptor prior to invading the host RBC [299, 302–304]. Complicating the situation, in the ensuing years, through research in multiple locales, it has become apparent that *P. vivax* can also invade Duffy negative RBCs by utilizing alternate invasion mechanisms (reviewed in [305, 306]).

Discovery of candidate vaccine proteins and testing them continues, predominantly to develop *P. falciparum* vaccines [279, 282, 284, 290, 295, 300, 307, 308]. Nonetheless, *P. vivax* research has been progressing at a competitive pace, even prior to the genome sequencing era, greatly aided by the use of both *Aotus* and *Saimiri* monkey infections and short-term in vitro blood-stage cultures permitting studies on *P. vivax* from human infected blood samples [303, 309, 310]. Over the span of several decades, these animal models have been critical for obtaining *P. vivax* parasites for genetic and biological analyses (reviewed in [19, 20, 311]), especially in light of the absence of a reliable and robust long-term blood-stage culture system for *P. vivax* [312–314], as has been available for *P. falciparum* for decades [315]. From a vaccine development standpoint, such research—and the use of the related *P. cynomolgi* and *P. knowlesi* parasites for fundamental basic biological studies and to generate crossreactive antibody reagents and perform basic experiments—has been critical for the identification of immunogenic merozoite surface and apically located proteins, which have been regarded as vaccine candidates (reviewed in [19, 25, 161, 299, 301, 311]). These include the PvDBP and the Reticulocyte Binding Protein (PvRBP) family members that have been shown to adhere to reticulocytes [300, 316–320]. Specifically, young CD71+ and CD98+ reticulocytes were identified as the host cells for *P. vivax* [13, 318, 321, 322] (reviewed in [323]). Importantly, the PvRBPs are related to the subsequently discovered *P. falciparum* Reticulocyte Binding Protein homologues (Rh) [324–326] and the Rh5 family member [327] has progressed from pre-clinical trials in *A. nancymaae* monkeys to clinical vaccine trials in humans [328–331]. Several of the above-named *P. vivax* proteins have been investigated as prospective vaccine candidates in field immunogenicity studies [332, 333], and several of these protein transcripts, along with others, were selectively upregulated according to comparative transcriptomic analyses of *P. vivax* infections in *S. boliviensis* and *A. nancymaae* monkeys, thereby pointing to roles in typical or alternative invasion pathways [334]. Identifying possible effective vaccine targets, for killing of parasites, remains a major goal.

An overarching challenge in the malaria vaccine development pipeline has been to express recombinant proteins or make peptides that can be representative of high-priority target proteins, structurally and antigenically, and on a population scale. While ongoing studies continue to establish a basis for new directions for malaria vaccine development, they are just the beginning. For example, a recent study assessed the global diversity and population structure of high priority *P. falciparum* vaccine candidate antigens [7]. This analysis included data from over 2600 parasite genomes from 15 malaria endemic countries and evaluated and compared the target haplotypes and 3-dimensional structures. Such analyses will help to optimize vaccine constructs to achieve broader efficacy in populations. Furthermore, protein microarray technologies have been used strategically and effectively to identify immunogenic parasite proteins from human and NHP samples [155, 290, 335], and creative genetic tools and parasite screenings are bearing new fruit [336–338]. The post-genome sequencing era with ‘omics’ experiments and ‘big data’ analyses continue to promise incomparably richer and more comprehensive understandings of parasite genes, transcripts, proteins, and metabolites, which together will hopefully reveal new promising high-priority vaccine targets. NHPs are important in this effort.

### Whole organism vaccine strategies

By the turn of the twentieth century, the prospects of whole organism malaria vaccines took root with both irradiated and genetically attenuated sporozoite immunization approaches, followed by chemoprophylactic regimens in combination with sporozoite immunogens (reviewed in [282, 308, 339–341]). These whole parasite vaccine approaches flourished despite many challenges and much to the credit of the company Sanaria™ successfully establishing necessary feasibility and ethical arguments [278]. As would be expected, whole parasite vaccines give broader immunity than subunit antigen or epitope-based vaccines [342], and *P. falciparum* sporozoite immunogens have shown homologous protection in malaria naïve individuals [50, 123]. However, the general efficacy of pre-erythrocytic vaccines in individuals in endemic areas has remained low (reviewed in [8]). Thus, the prospects for their widespread use in malaria endemic areas have been in question, raising new challenges to be overcome, largely around the need to better understand NAI and VCI responses in individuals with prior and likely repeat exposure to *Plasmodium* infections and malaria. By contrast, *P. falciparum* sporozoite immunizations along with prophylactic drugs have shown homologous and heterologous efficacy and have been suggested for use by travelers to Africa and potentially widespread use in Africa [343–345]. Comparable research on *P. vivax* sporozoite vaccines has yet to be reported. This research has been hampered by the unmet challenge of reliably generating sporozoite immunogens in the absence of blood-stage culture systems that can reliably produce infectious gametocytes for infecting *Anopheles* mosquitoes. A few studies with sporozoite immunizations concurrent with blood-stage drug cover have been carried out with *P. knowlesi* [342, 346] and *P. cynomolgi* in *M. mulatta* ([347] and Joyner et al. pers. commun.), providing groundwork for future experiments where NHP model systems can delve into mechanisms at work in the face of these strategies.

### Major impediments to immune intervention, challenges and opportunities

Now, in the twenty-first century, the lack of detailed understanding of NAI against malaria caused by any species and its limited effectiveness such that chronic parasitaemia ensues rather than sterilizing immunity, and the general lack of reliable malaria vaccine correlates of protection, have been recognized as major impediments to the elimination and eradication of the disease through vaccines or other immunologically based interventions (reviewed in [6, 8, 35, 96, 348]). It is also unclear how chronic exposure and immune activation to any species of *Plasmodium* impacts the establishment of new immunity against specific antigens represented in vaccine candidates. Specifically, chronic exposure to *Plasmodium* infection may interfere with inducing efficacious protection, as evidenced recently in malaria endemic field-based vaccine trials (reviewed in [8]). Moreover, economic and cultural factors may contribute to poor nutrition and baseline poor health with anaemia being common in malaria endemic areas, or incomplete intake of costly anti-malarial regimens can result in chronic infections and the potential for ongoing transmission. With progressive understanding of immunological mechanisms, networks, and cascades, novel vaccines and other immunological-based interventions may be envisioned that, ideally, will more successfully target and eliminate the infectious parasite load, thereby supporting host resilience and recovery in malaria endemic areas. Some ideas in this direction have been discussed in relation to the finding that pre-immunization inflammation was associated with malaria vaccine protection [49].

#### Innate and adaptive immunity

In brief, innate immune signaling pathways are activated by parasite-derived materials such as DNA, RNA, glycosylphosphatidylinositol (GPI), and haemozoin, otherwise known as Pathogen Associated Molecular Patterns (PAMPs) or Danger Associated Molecular Patterns (DAMPs) (reviewed in [349–352]). Subsequent adaptive responses target infected RBCs and help suppress clinical disease. Much remains to be understood about the innate responses attributed to various cells including γδ T cells, natural killer cells, NKT cells, dendritic cells, monocytes/macrophages, neutrophils, and platelets [353–355], and adaptive B cell and T cell responses, including memory responses, and the relative importance and host dependence upon each [356]. Likewise, the importance of various immunoglobulin isotypes remains to be fully understood [357, 358]. Importantly, IgM has become recognized in human clinical studies as relevant for protection against disease caused by *P. falciparum* during both acute and chronic infections, being produced initially upon infection as well as through memory B cell (MBC) responses [359–363]. Long-lived IgM responses were detected against 15 antigens in recent *P. vivax* immunogenicity field studies, raising questions on the origin and role of IgM compared to IgG [364, 365]. Also of note, a recent transcriptomic study found that genes and networks associated with ubiquitin-proteasomal proteolysis, which are important for innate and adaptive immunity, were disrupted in children with severe malaria [366].

CHMI experiments have served as an alternative to traditional field studies by attempting to explain the immune response to malaria parasites over time in malaria naïve and non-malaria naïve individuals. For example, one noteworthy observation is that epigenetic imprinting occurs in innate immune cells after exposure to blood-stage parasites in vivo [367]. This is interesting given recent advances generally on the potential importance of innate immune memory, including against *Plasmodium* [368, 369], and “trained immunity”, defined as heterologous immune responses elicited by live vaccines [370]. It remains unclear how much trained immunity responses are relevant to NAI against malaria. Also of consideration, during CHMI studies initiated with sporozoites, treatment is given when parasitaemia is first detected (i.e., when low), and therefore, the ‘natural’ parasite antigen load and inflammatory responses will be reduced, thus altering the natural course of immunity.

#### Disease, tolerance and recovery

A hallmark of acute malaria is an array of inflammatory processes that can cause fever and other symptoms and signs of illness, which result in possible mild to severe damage to the body’s tissues and organs. Inflammatory processes can aid a host in its anti-malarial fight and recovery, but they can also overwhelm the host [371]. Numerous known and yet-ill-characterized immunological mechanisms contribute to disease tolerance, for example, involving control of monocyte/macrophage activation [369] and peripheral blood mononuclear cell (PBMC) regulatory [372] responses, and enhanced p53 expression in monocytes that was shown to attenuate inflammation and predict protection from fever [373]. Future research is warranted to better understand these and other such processes, which can suggest possible host-directed and adjunctive therapies that may support host wellness and survival.

#### Asymptomatic infections, chronicity, and anaemia

The majority of human *Plasmodium* infections are asymptomatic with chronic long-lasting infections that provide a blood-borne reservoir of parasites for transmission to *Anopheles* mosquitoes and propagation of the disease [374, 375]. Unless active case detection measures are in place, these asymptomatic infections generally go unnoticed and remain a major barrier for malaria elimination and eradication efforts. This fact raises a key question: Why is the immune system unable to rid the body of all parasites? Future research should include focused efforts to identify the parasite’s immune evasion targets and mechanisms. Additionally, the expansion of so-called atypical memory B cells (AtMBCs) has been reported in people chronically exposed to malaria parasites [376–379]. These cells are characterized by the expression of inhibitory markers, and they can be activated by inflammatory signals, such as IFNγ and TLR-9 activation by parasite DNA (reviewed in [380]). Recent data suggests that AtMBCs or IgM producing MBCs may be part of a normal memory immune response in chronically exposed or vaccinated individuals and various questions remain relating to their development and functionality [359, 363, 380–382]. In in vitro co-cultures, AtMBCs increase and classical memory B cells contract in the presence of parasites [383].

Future studies using NHPIMs can help advance this critical line of research by determining from which B cell tissue compartments these cells originate, the extent of their persistence, to what cytokines they respond, and to the extent they help or hinder the development of immunological memory responses against the parasites [384]. Delineating their contributions to parasite neutralization as well as pathogenesis is critical in part since AtMBCs are known to produce anti-erythrocyte antibodies that can contribute to the destruction of both infected and uninfected erythrocytes [385–387]. These data expand upon prior knowledge relating to autoimmune or specific anti-erythrocyte antibodies in the pathogenesis of malarial anaemia [388–393]. Future goals should include tracing these responses between the tissues and the peripheral blood, especially to enhance understanding of immune memory provided by T and B cells, but also since a preponderance of immune activity occurs in the tissues and the peripheral blood provides a limited view of the systemic activities.

#### Immunosuppression

In 2013, Mueller and colleagues reviewed known information relating to *Plasmodium* infections causing immunosuppression, including the influence of regulatory T cells, B cell populations, and antigen presenting cells [25]. This topic is of high interest and was expanded upon in 2021 by Calle and colleagues [394]. As immunosuppression is evident during acute infections and afterwards, and can persist during chronic infections, they have stressed the critical points that: (1) malaria impacts the overall health of the immune system, and (2) it is important to conduct research aimed towards reestablishing normal functions. This is generally important for public health but also to support the development of effective immune responses to vaccines.

#### Route to interventions

Decades of immune response studies converge on similar conclusions with regards to the need to understand much more about the intricate host–parasite relationships and immune responses that allow for sustained parasitism [19, 46–48]. Such knowledge has the potential to reveal new immunological targets of intervention, whether against the parasite, or in the form of host-directed or adjunctive therapies, including repurposed drugs [395, 396]. A greater understanding of the NAI responses that lead to partial (or possibly complete) protection is needed, as well as in-depth knowledge about the host and parasite receptor-ligand target molecules required for successful host cell invasion and parasitism, the immune cell types, factors, and pathways that become activated to ward off an infection, the parasite’s immune evasion tactics to overcome such immune activity, and the cascade of pathological consequences that follow.

Normal physiology is affected by malarial infections and tissues can become damaged; in severe cases organ damage may be irreparable. These facts are also evidenced from rare cases of irreversible severe illness caused by *P. coatneyi* when modelling *P. falciparum* infection [129] and *P. cynomolgi* modelling *P. vivax* infection [397]. Key questions include why individuals living in malaria endemic countries do not develop sterilizing immunity after one or a few infections, and why premunition is not long lasting. An overarching question is why immune memory is insufficient to completely ward off or eliminate new infections. As discussed above, the human host does not completely lack immune cell memory recall responses, but that they are complex and multi-faceted, with many unknowns. Humans also face diverse infecting parasite species and strains, which makes infection scenarios much more complex yet nonetheless akin to what the world has been experiencing with the spread of SARS-CoV-2 variants [398, 399]. The lack of complete immunity is also predictably due to evolutionary safeguards to ensure parasitism, including immune evasion strategies, such as antigenic variation (reviewed in [92, 400]) and escape mechanisms that ensure gametocytogenesis (reviewed in [400–402]). Specifically, while infected hosts mount primary and memory recall responses, they tend to favour (*or are restricted to achieve*) a reduction in parasitaemia and illness over elimination of all parasites, therewith supporting the retention of sexual stage gametocytes and prospects for transmission to mosquitoes.

Systems-based studies of the immune response and cellular dynamics during longitudinal infections will continue to be informative to help address long-standing questions and arrive at new interventions. While clinical immunity can be generated, resulting in asymptomatic infections, chronic sub-patent blood-stage infections remain the norm in endemic areas. This situation naturally serves the parasite well, allowing for transmission of gametocyte progeny to mosquitoes, but remains a health concern for infected individuals as well as the population at large that presents targets for future bites by mosquitoes and propagation of the disease. If natural inoculation of the parasite itself does not lead to sterilizing immunity, what are the chances that a subunit or whole parasite vaccine would? The often-presumed answer is that sterilizing immunity is unlikely, or an extremely elusive goal [96], regardless, and this conclusion has so far been born out in vaccine challenge studies in malaria endemic areas (reviewed in [8]). Aside from vaccines leading to specific protein-targeted immune responses and in general perhaps overcoming parasite strain diversity and antigenic variation, they may need to conquer strain-transcending immunity, although this is still ill-defined [35, 403].

## Malaria research advances with systems approaches and nonhuman primate infections

### Host–parasite interactions: systems biology, immunology, and vaccinology

A complete understanding of malaria parasite species, their biology, host–parasite interactions, and population dynamics that are essential for their survival, is still lacking. In 1971, at the time of the publication of “The Primate Malarias”, host–parasite interaction(s) was not common terminology [404, 405]. This terminology has taken hold in the post-genome sequencing era, over the past 20 years, where “functional genomics” and the integration of big data sets have become the tools of computational biology research teams [406, 407]. Still today, as was the case 50 years ago, scientists often stand at the cusp of entering deep into the abyss of the unknown, whether aiming to reveal host–parasite receptors, intra-cellular development and growth mechanisms, parasite evasion mechanisms, or to gain a solid understanding of the cascades of molecular and cellular events that result in both disease pathogenesis and immunity. This is palpable when using systems biology, systems immunology, or systems vaccinology approaches (reviewed in [8, 46–48, 294, 407–411]). Systems biological and immunological approaches are now being used to help interpret and integrate genomics and associated multi-omic data, with the goal of enabling a more holistic if not comprehensive understanding—and predictive capabilities—of the multitude of host–parasite interactions and biological pathways that have evolved to allow successful parasitism: with parasite infection and evasion strategies functioning alongside host immune defense mechanisms. Likewise, systems vaccinology aims to incorporate and visualize high-dimensional data to understand VCI responses and outcomes [412].

Whole blood transcriptomics has been a starting point for numerous functional genomics projects. Transcriptomic models—especially across multiple time points from in vivo infection—gain multi-dimensional complexity when epigenetic interactions and time-dependent biological and immunological phenomena or the impact of the microbiota or co-infections are considered as well (reviewed in [46–48, 410]). By integrating transcriptomics and various datatypes, Tran and colleagues identified multiple immune signatures including p53 upregulation in children in Mali that associated with their tolerance to blood-stage infection and fever [373]. Transcriptomics and metabolomics data were integrated by Gardinassi et al. from a *P. vivax* CHMI trial to define specific immune responses [484], and by Cordy et al. to define metabolic signatures associated with acute and chronic disease caused by *P. coatneyi* and *P. falciparum* [131]. Gupta et al. recently used systems approaches, including transcriptomic and metabolic modelling, to show distinctive relationships between longitudinal *P. knowlesi* infections in *M. mulatta* and *M. fascicularis,* which highlight mechanisms of resilience in the latter species [396, 413]. Systems vaccinology approaches were used to study an RTS,S candidate vaccine, demonstrating NK cell responses as correlates of protection associated with RTS,S vaccination [414]. Recently, systems-analysis of multiple malaria vaccine trials has shown that pre-immunization inflammatory responses correlate with protection [49]. Baseline data have also been modelled and integrated to provide predictive information regarding the immune response, as shown for example with an influenza vaccination study that evaluated the frequencies of PBMC subpopulations, the results of which the authors suggest may in part reflect influence by the microbiota or other infectious agents [415]. Pre-immunization correlates of protection have in fact been determined for multiple and diverse vaccine types: against influenza, hepatitis B, yellow fever, and malaria (reviewed in [49]). These examples and many others [46] raise the prospects for discoveries that may improve efficacy of vaccines, by stimulating innate responses predicted to confer protection. However, human trials for malaria vaccines and other diseases have shown that not only internal responses but external environmental factors including endemicity and prior exposure to diseases greatly impact immunity and the ability to predict outcomes of vaccines based on testing in naïve individuals alone (reviewed in [8, 57, 294]). Thus, major challenges remain, at many levels. These include the importance of understanding NAI and VCI responses in children compared to adults, and with different levels of previous exposure to malaria. Such basic research is crucially important, as recognized for many infectious diseases, including SARS-Cov2/Covid-19 [416, 417].

The predictive capabilities, based on mathematical models and the integration of diverse datasets and metadata, support the identification of signature biomarker profiles that may be prognostic or diagnostic, validation experiments that may include perturbations, and in silico screening and testing of candidate drugs and vaccines. Traditional ELISPOT tests, ELISAs, isotype analyses, protein microarrays, and various functional assays (e.g., phagocytosis assays and parasite inhibition of invasion tests) continue to serve a purpose, to validate findings and develop new hypotheses and research directions to delve deeper into the biology of specific cell types and immune networks. Meanwhile, bioinformatic analysis of large and multi-omic datasets, dynamic modelling and spatial imaging technologies are becoming critical for advancing such research, whether on single cells or tissue samples (reviewed in [219, 221]). For an overview of the processes that comprise systems biology, from big data analysis to its visualization to building of complex models and reductionist experimentation to validate the models, various articles are recommended [406, 407].

The integration of multiple-omic and other data types is a key goal for designing quasi-realistic computational models for biological processes. In the simplest case, these are static representations of networks that show which components interact with each other. Dynamic modelling raises the bar further by also addressing questions pertaining to the constant changes in biological systems, such as a host cell or organism, whether these occur within seconds, minutes, a day or longer [418, 419]. Generically, it is hoped that systems approaches will flesh out knowledge gaps in key areas of host–parasite interactions. Pursuing this avenue, new basic, preclinical, and clinical research needs are being defined and prioritized with a burgeoning number of concerted efforts involving multidisciplinary and transdisciplinary teams of researchers. All these modelling efforts require very large quantities of data, and these are beginning to emerge in public databases that offer access to large, system-wide datasets for reuse and iterative analysis (reviewed in [8]). A prominent example for malaria research is PlasmoDB [420, 421], which is associated with the MaHPIC’s longitudinal NHP experiments, described below. While mathematical modelers in the past lacked data, now there is an overload. This has created the need for biologists and mathematical and computational experts to interact, partner, and rely upon each other.

### Genomics data are available for key host and parasite species

Genomic and post-genomic technologies have greatly advanced knowledge of malaria parasites of a number of species in various stages of their life cycles. The genome sequences of *P. falciparum* and three rodent malaria parasite species were tackled first, at the turn of the century [14, 422, 423], followed by others (mentioned below) and recently six Great Ape *Laverania* malaria parasite species [424] plus a fourth rodent malaria parasite species [425]. This species priority list was largely based on *P. falciparum* causing the most cases of malaria throughout Sub-Saharan Africa, including severe cases and death, and because *P. falciparum* and corresponding rodent species are recognized as being most widely amenable to research work in laboratories around the world. However, the in vitro culture environment is very different from the in vivo host environment, with all its complex organ physiology, biology, biochemistry, and immune system, and rodent models—while valuable—do not suffice to reveal the host–parasite interactions that occur in humans or NHPs [426, 427]. Particularly given the availability of the first and additional *P. falciparum* genomes and robust culture systems, functional genomics studies have become plentiful for *P. falciparum* for the different parasite development stages throughout the parasite’s life cycle [176, 410, 428].

*Plasmodium vivax* and *P. knowlesi* genome sequences were published in 2008 [429, 430], followed by additional *P. vivax* [431–433] and *P. knowlesi* [434–437] genome sequences including the use of advanced technologies and clinical isolates. *Plasmodium vivax* transcriptomes have since become available representing sporozoites [438, 439], specific liver-stage forms including hypnozoites [212, 213] and blood-stage forms [440–448], and proteomes have been developed representing stage-specific blood stages and clinical biomarkers [449–454]. Likewise, transcriptomes have been reported for *P. knowlesi*, importantly showing differences in the parasite’s gene expression between the in vivo NHP environment and in vitro cultures [455] as well as differences in *M. mulatta* and *M. fascicularis* host responses [396]. *Plasmodium knowlesi *ex vivo stage-specific blood-stage proteomes are under analysis, helping to flesh out the biology of this species (Anderson et al. pers. commun.). *Plasmodium cynomolgi* genome sequences were first published in 2010 [186], followed by an improved genome [168] and transcriptomes representing stage-specific LSFs [208, 214]. Finally, the *P. coatneyi* genome sequence was published in 2016 [254] and in 2016 and 2017 genome sequences were reported for the remaining two human malaria parasites, *P. ovale* and *P. malariae* [15, 456]. Human and NHP (*M. mulatta*, *M. fascicularis* and *Aotus* and *Saimiri* species) genome sequences have also been published [457–463]. So, the genomic puzzle pieces are available to study the interactions of several key parasite species in the context of their human or NHP hosts and, in some instances, with host cells in ex vivo or in vitro experiments. This is in addition to the completion of the genomes of *Anopheles* vector species and ongoing advancements for studying *Plasmodium* genomics data [464].

Still and all, the biological functions of most *Plasmodium* proteins remain undefined, but expedited progress with biological discoveries are likely given the immense amounts of genomic and functional -omic data that continues to be developed and analysed. Technological tools and capabilities have advanced considerably to obtain genomic sequences with improved quality and much more readily from unknown parasite strains, as well as -omic information on host tissues and single cells [465], and when accessible, spatial data providing the interactive physiological tissue and cellular context of both [221]. Today, with advances in big data technologies that enable the generation and analysis of -omic and other large-scale complementary clinical datasets from small blood volumes (100 µl or less, and even from dried blood spots), human blood samples have become amenable to systems biological research that can complement basic in vitro studies and research involving rodents or NHPs.

### Deciphering malaria with the support of animal models

The malaria research field and its vision for what’s important and possible have advanced considerably with the recent benefits of CHMI and systems biological and immunological approaches, generating new levels of understanding and numerous new hypotheses. Still today, underlying biological and molecular mechanisms of NAI, VCI and pathogenesis are largely unknown, leaving a hazy picture of the immune response under various scenarios with many gaps and open questions [46, 47]. However, immunological tools and technologies are advancing to make new discovery in these areas possible, and now tantamount, even with chronic infections [375]. Future research is bound to expose the inner workings of many critical biological, immunological, and pathogenic mechanisms and pathways, including immune evasion strategies, and both rodent and NHP animal models can support such endeavors. To the extent possible, it is important to know infection history, lifetime exposure, and how long an individual has been infected. Huge strides cataloguing such essential data have been made by a few research groups performing clinical studies with longitudinal sampling that includes repeat sampling from the same individuals [232, 233, 373, 466–468]. Unfortunately, these types of studies are labour intensive and arduous, and not typically possible to complete on large scales.

Numerous investigations for decades have relied upon rodent malaria models to delve into immune response and pathogenesis questions (reviewed in [426, 427]). Apart from expensive model systems such as humanized mice, rodent models are economical and practical, including affordable per diem rates to house and feed the animals and the ready availability of technical support for working with these animals. Moreover, the ability to mimic major clinical manifestations of malaria such as anaemia, cerebral malaria, acute kidney injury, and chronic infection using selected rodent malaria parasite species in conjunction with selected host genetic backgrounds and genetically manipulated parasites have made investigations using rodent model systems efficient and reproducible, alongside studies to decipher the function of the many uncharacterized parasite proteins expressed at different stages of the life cycle (reviewed in [422, 425, 469]). Collaborative cross mice [470, 471] may also serve to identify genetic backgrounds that support the development of chronic, asymptomatic parasitaemia, but significant differences compared to humans and NHPs regarding their immune systems and physiological responses remain, as do other differences and associated limitations. Thus, the trade-off for the utility and reproducibility using rodent models will remain a loss of the complexities and intricacies of malaria pathogenesis that is clear from observations on malaria in humans and NHPs.

NHP infection models (NHPIMs) allow for the in-depth investigation of both broad overarching and specific hypotheses, providing opportunities to enhance what can be achieved using rodents and through clinical studies with humans. Ideally, research using NHPs will continue to complement or validate studies involving rodents, where putative mechanisms can be investigated in greater depth with the benefit of genetic tools and much larger experimental cohorts. Still, rodents can develop exceedingly high parasitaemias, well over 10% in most cases, which undoubtedly must affect immune responses. Such a high level of parasitaemia is rarely seen in human infections or carefully monitored and controlled NHP infections. Moreover, relapse biology and associated immune responses currently cannot be studied using rodent malarial infection models (particularly given the lack of relapses in rodents, except in humanized mice [472, 473]), and as discussed above research on relapses is formidable with humans [231–233], making the NHP models for studying relapses preferable if not essential.

In many cases, immune cell subsets between humans and macaques are comparable. Peripheral blood B cell subsets and T-cells can be phenotyped and classified similarly between humans and macaques [139, 474, 475]. Other cell types can also be purified and studied, whether singly or as populations. These strengths of NHP macaque models are further enforced by the ability to access tissues longitudinally or in terminal studies along with peripheral blood. In rodent model systems, tissue samples can also be obtained, but only very small blood volumes are available, limiting determinations on how immune responses in the tissues relate to changes detected in the peripheral blood. Coupling the strengths of macaque models with the many available species and strains of simian malaria parasites makes macaques especially well-suited for immunological investigations, and particularly for systems-based longitudinal studies because of the reasonable amount of informative sample materials that can be obtained over time, covering the entire course of the disease from pre- to post-infection scenarios, and possible re-infection or co-infection situations [476–480].

The time is opportune to maximize NHPIM efforts, building upon experience gained from longitudinal NHP experiments by many investigators over many decades (described above), and recently by the MaHPIC, described below. Incidentally, the MaHPIC was established soon after the publication of a review article, titled “Acquired Immunity to Malaria”, where the authors, Doolan, Dobaño and Baird wrote: “One likely starting point for more firmly establishing age-dependent, strain-transcending immunity in malaria and for beginning to sort through the myriad possible mechanisms at work may be a nonhuman primate model” [35]. Similarly, Mueller et al. concluded: “In addition, a more thorough use of the existing non-human primate models as well as experimental infection in human volunteers will be essential for advancing our understanding of the basic processes and specific antigens involved in the establishment of NAI to *P. vivax* [25].” As noted by Doolan and colleagues, by 1920, the essential elements of NAI had been described. In essence, natural immunity is: “(1) effective in adults after uninterrupted lifelong heavy exposure, (2) lost upon cessation of exposure, (3) species specific, (4) somewhat stage specific, and (5) acquired at a rate which was dependent upon the degree of exposure” [35]. Also, Lefebvre and Harty have emphasized the importance of NHP infections for studying protective immune responses in the liver [96], also indicating that needle aspirate techniques may support such efforts [481]. Silva-Fila and colleagues further recognized the value of both CHMI and NHP research to address immunity and pathogenesis questions, with emphasis on the importance of accessing bone marrow samples [97].

## Establishing academic environments for NHP research with complete life cycles

Between 1999 and 2019, the Yerkes NPRC (YNPRC) at Emory University in Atlanta, Georgia (in 2022, renamed as the Emory National Primate Research Center) became recognized as the main NPRC (in the USA) with malaria as a major area of research emphasis, and with a recent history of systemic longitudinal malarial infection studies and an on-site insectary facility. Other academic institutions offer the possibility for NHP studies, or mosquito work, but rarely are these animal and mosquito resources found together in one location, so that NHPs can become experimentally infected via mosquito bites or the inoculation of freshly dissected sporozoites from the salivary glands of mosquitoes. The Yerkes Insectary for Malaria Research (YIFMR) was designed in 2015 and opened in 2017, in response to the Centers for Disease Control and Prevention (CDC, Atlanta, GA) plans to eliminate malaria research projects that involved NHPs. Thus, there was the need to fill this gap, building upon the CDC’s renowned basic infection and evolutionary studies using a variety of primate malaria species and NHP hosts to document host–parasite compatibilities, vaccine trials, and the routine production of gametocytes for infecting female *Anopheles* mosquitoes to obtain infectious sporozoites (reviewed in [11, 12]). Up until 2017, the CDC was the source of mosquito salivary gland-derived infectious sporozoites for Emory’s malaria research team, including for the MaHPIC, in support of a series of published and unpublished longitudinal infection experiments (described below). The YIFMR was designed for rearing multiple species of *Anopheles* mosquitoes—for infections with various *Plasmodium* species of interest (i.e., *Anopheles dirus*, *Anopheles gambiae, Anopheles freeborni*, *Anopheles stephensi*)—and infecting them through direct blood meal feedings on NHPs.

Comparable setups have since been established at two other academic institutions in the United States: the University of Georgia (Athens, GA, currently directed by Chester J. Joyner) with New World and Old World monkey resources and the Oregon NPRC (Portland, Oregon, currently directed by Brandon Wilder) with Old World monkey resources. The expansion of such research is important, especially now while there is potential for in-depth exploration of the life cycle, immunity and pathogenesis using systems biology, immunology, and vaccinology approaches.

### The Malaria Host–Pathogen Interaction Center (MaHPIC): building infrastructure for addressing malaria in a systemic manner

Malaria is so complex that systemic analyses have become critical, and these require large transdisciplinary teams that encompass many scientific disciplines. The Malaria Host–Pathogen Interaction Center (MaHPIC) is an example of such a large team effort, which has been focused primarily on NHPIMs and using systems approaches to develop and integrate multi-omic, clinical and parasitological datasets from longitudinal infections. As of the time of this writing, no comparable research program has been noted in the literature, and as discussed above, these studies complement CHMI investigations.

The MaHPIC was established in 2012 at the YNPRC of Emory University [482] and conducted a series of longitudinal NHP infection experiments through September 2017 with USA contract support from the NIH’s National Institute of Allergy and Infectious Diseases (NIAID). The MaHPIC’s founding team comprised investigators from Emory University, the University of Georgia in Athens, the Georgia Institute of Technology, and the CDC, as well as collaborators involved in clinical studies from around the world ([131, 483, 484], and Cordy pers. commun.). The team included malariologists, biologists, biochemists, immunologists, pathologists, clinicians, veterinarians, and mathematical, computational and informatics experts. By September 2017, about 14 terabytes of MaHPIC data had been generated and ﻿the majority was released to public websites [482, 485, 486]. Between 2016 and 2019, the team’s work was complemented by the U. S. Defense Advanced Research Projects Agency (DARPA) with funding for a project called “THoR’s HAMMER” (Technology for Host Resistance, Host Acute Models to study Experimental Resistance). Here, the main goal was to reveal characteristics of malarial resilience, by comparing *Plasmodium* infections in multiple host species, which showed either severe or resilient outcomes (see experiments called E06, E07, E30, E33 and E35 below).

The MaHPIC team put forth the overarching hypothesis that “Nonhuman primate host interactions with *Plasmodium* pathogens as model systems will provide insights into mechanisms as well as indicators for human malarial disease conditions”. The MaHPIC’s work has been built upon three underlying scientific premises: (1) that the host environment is critical for studying malaria parasite biology, infections, immunity, pathogenesis, and pathology, (2) that studying the dynamics of longitudinal infections is critical, and (3) that the integration and modelling of parasitological, clinical, and multi-omic datasets will reveal novel host–parasite interactions, pathways, and networks.

The MaHPIC’s primary mandate from the NIAID was to run longitudinal infection experiments in macaques, collect peripheral blood and bone marrow samples at specific time points, and generate clinical, parasitological, and multi-omic datasets, which could then be analysed and integrated—initially by the MaHPIC team, and subsequently by others. Representative MaHPIC longitudinal infection experiments and key results are summarized below. Parasites used in the MaHPIC’s experiments included *P. coatneyi* (Hackeri strain) as a model of *P. falciparum*, *P. cynomolgi* (M/B and Ceylon strains) as a model of *P. vivax*, and *P. knowlesi* (Malayan strain) to better understand malaria caused by this zoonotic species in its natural (human and *Macaca fascicularis*) and the traditional experimental (*M. mulatta*) hosts. Several infection experiments were also carried out with *P. vivax* in *A*. *nancymaae* and *S. boliviensis* monkeys. Each major longitudinal NHP infection was initiated with sporozoites and allowed to progress for over one month, and in many instances three or more months, when repeat homologous or heterologous sporozoite-initiated infections, relapsing malaria, or recrudescing malaria and chronic disease were being explored. Typically, capillary blood samples were obtained daily as warranted for parasitaemia readings and reticulocyte count analyses, blood chemistry readings using an iSTAT System (Abbot Labs), and targeted and/or untargeted plasma-based metabolomics experiments. Designated time points for venous blood draw samples were determined and selectively utilized as required to generate various large-scale-omic datasets (transcriptomics, metabolomics, lipidomics and/or proteomics), cytokine analyses, serology, adaptive and innate immune profiles, complete blood count haematology measurements, and the enumeration of haemozoin-containing leukocytes. The details of sample collections, methods, and data generated are being compiled elsewhere (DeBarry et al. manuscripts in preparation), in addition to related details already reported with MaHPIC’s public database depositions and in the team’s original research publications (listed at [482]). This body of work represents the ‘tip of the iceberg’ when considering what is possible.

The MaHPIC’s original mandate did not include investigations of the immune response in depth, but the team increasingly built the foundation for doing so given the immune response’s prime relevance to infection and disease. At the start of the MaHPIC, a few basic innate and adaptive immune profiling flow cytometry panels were included in the project’s experimental design, to enable initial comparative analyses of the dynamics of cell types that are activated in the course of infection by different malaria parasite species. The group’s foray into malarial immunity and pathogenesis gained increasing attention, and the design of major infection experiments shifted to address specific outstanding biological, immunobiological, and pathogenesis questions of importance to the field of malaria research. As a result, NIAID also awarded the team supplementary funding to design and implement a systems vaccinology trial experiment, specifically to examine the immune response in *M. mulatta* to *P. cynomolgi* sporozoite immunizations in the face of blood-stage drug cover and the outcome from challenging the animals with infectious sporozoites (Joyner et al. pers. commun.).

In total, the MaHPIC successfully optimized an extensive number of flow cytometry panels and gating strategies to track B cells, T cells, monocytes, dendritic cells, neutrophils, and NK cells, as well as cell activation, co-stimulation and trafficking functional markers when relevant. RBC flow cytometry panels and procedures were also developed to monitor erythrocyte progenitor cells, different stages of RBC development from nucleated RBCs to reticulocytes and normocytes, and to distinguish uninfected and infected RBCs [92]. 45 cytokines were identified using Luminex assays and 1300 proteins including among them cytokines were quantitatively measured using SOMAscan—an aptamer-based targeted proteomic technology (SomaLogic, Inc.; Boulder, CO, USA) [487]. Fundamental work was designed to assess the various immune cell populations and specific subsets in the peripheral blood and bone marrow at baseline time points and at specific later time points throughout the course of each longitudinal infection experiment with *P. cynomolgi*, *P. coatneyi* or *P. knowlesi*. Special flow cytometry panels were developed to track and evaluate specific cell types in *A*. *nancymaae* and *S. boliviensis* involved in the immune response to *P. vivax*. As a result, numerous fluorescently labelled antibodies were identified, tested, and validated in MaHPIC experiments (DeBarry et al. manuscripts in preparation).

### Selected longitudinal infection experiments, performed by the MaHPIC

Iterative longitudinal infection experiments were executed by the MaHPIC. A few representative examples are summarized here to demonstrate the types and results of these experiments. The experimental numbers reflect those incorporated in the group’s Laboratory Information Management System.

#### Experiment 3 (E03): *Plasmodium coatneyi* infection of *M. mulatta*

The MaHPIC’s initially designed longitudinal infection experiment (called E03), involved repeat infections of four malaria naïve *M. mulatta* with 100 *P. coatneyi* sporozoites [131]. This was designed to build upon knowledge gained in a blood-stage longitudinal experiment focused on anaemia [130]. Then, it was confirmed that the malaria naïve *M. mulatta* developed severe anaemia, coagulopathy, renal impairment, and a generalized metabolic dysfunction comparable to what is often seen in severe human cases. Major turnover of erythrocytes was demonstrated and attributed to dysfunctional bone marrow. Disease severity turned out to be less once the animals were semi-immune and re-infected [130].

Specifically, E03 [131] showed that rhesus macaques infected with *P. coatneyi* develop chronic infections after receiving subcurative artemether treatment and that they recapitulate the decrease in haemoglobin levels characteristic of persistent infection in humans [45, 488]. A recent study with kra monkeys infected with *P. knowlesi* showed evidence of a similar phenotype, but without the administration of subcurative anti-malarial treatment (see E07 below) [268]. Both situations—drug treated or not—are highly relevant for performing comparative systems-level analyses of host–parasite interactions. The E03 study showed specific transcriptomic and metabolic changes associated with acute and chronic infections [131]. Furthermore, distinct metabolic profiles were identified in *M. mulatta* that were comparable to those observed in human *P*. *falciparum* cases. E03 data are still being analysed by the MaHPIC team, for instance, with respect to the integration of transcriptomic and immunological data. Of particular importance has been an analysis of the development of specific antibody responses over time, as chronic infections are established, and the dynamic production and function of different isotypes. A main question relates to how these antibody responses may result in a balance of protective responses with the killing of infected RBCs and adverse responses that result in the elimination of a majority of uninfected bystander RBCs, as determined by Fonseca et al. using a mathematical model [489].

#### Experiments 04, 23, 24 and 25: *Plasmodium cynomolgi* infection of *M. mulatta*

These four iterative experiments adopted the *P. cynomolgi*-rhesus macaque model of relapsing malaria to delve into specific immunobiological questions and begin the process of using systems biological approaches to understand relapse biology and anaemia as it relates to *P. vivax* in humans [490]. The course of infection and relapse patterns were studied in experiments involving 11 naive rhesus monkeys, which had been inoculated with 2,000 *P. cynomolgi* M/B strain sporozoites that had been freshly isolated from *Anopheles* mosquito salivary glands [139, 140]. The time to blood-stage infection was comparable in all animals, with patency reached within 10–12 days, and all animals relapsed in the course of the study (set for 100 days; initially with a group of five animals designated for E04, and then with six animals designated for E23, followed by repeat parasite inoculations in consecutive experiments called E24 [139] and E25 (Joyner et al. pers. commun); one animal from E04 needed to be euthanized due to severe malaria, as it was not responding to treatment [397].

Based on data from E04, Tang et al. used Weighted Gene Coexpression Network Analysis (WGCNA) to integrate flow cytometry, RNA-sequencing, and systemic cytokine measurements [491]. Despite anaemia and an increase in erythropoietin levels during the acute infection, the bone marrow did not respond appropriately to compensate for blood losses [491]. The acute infections showed ongoing inflammatory responses with Type I and Type II interferon transcriptional signatures, which were associated with intermediate and non-classical monocytes. The complete analyses suggest that, mechanistically, the observed insufficient erythropoiesis may be due to monocyte-associated disruption of GATA1/GATA2 regulation. In summary, monocyte-associated inflammation in the bone marrow influenced the disruption of GATA1/GATA2 transcriptional networks that are required for the differentiation of erythrocytes [491]. In contrast, anaemia was not observed during relapses and the bone marrow samples at that time did not show transcriptomic perturbations reflecting inflammatory responses and dysregulation of erythroid progenitor cells [491]. This is the first systems-level analysis based on NHP longitudinal infections that integrated multiple data types to identify a molecular mechanism that contributed to bone marrow dysfunction, and it provides direction for further study with a focus on longitudinal bone marrow sampling. Shortly thereafter, Brito and colleagues published experiments using bone marrow aspirates from naturally infected *P. vivax* patients and confirmed a similar mechanism in humans [447]. After decades of realizing that the bone marrow is affected by malaria parasites [492], the mechanisms are now becoming accessible. Additionally, MaHPIC members integrated transcriptomics, metabolomics, and lipidomics data from E04 peripheral blood and bone marrow samples, and synthesized them with immunophenotyping data using a unique and intuitive mutual information-based network analysis approach. This effort distinguished uninfected NHPs, acute infections and relapse infections, in an unsupervised manner, revealing information not possible with a single -omic data type [493].

Systems analyses are showing that macaques are particularly well-suited for investigating changes in the bone marrow and possible relationships to systemic changes impacting not only anaemia, but also the development of long-lived serological immunity. E23 and E24 demonstrated that—as with humans (shown with malariotherapy studies; reviewed above)—clinical immunity can form after a single sporozoite-initiated blood-stage infection and prevent illness; this was the case both during E23 relapses and E24 homologous strain reinfections [139]. For this study, along with clinical and parasitological data, data were integrated from whole blood RNA-sequencing transcriptomics, flow cytometry distinguishing B-cell subpopulations, *P. cynomolgi*-antigen-specific ELISAs, and opsonic phagocytosis assays to support the functionality of *P. cynomolgi* antigen-specific antibodies. Together, the various data types and validation experiments demonstrated that immunity after the primary parasitaemia and a bout of clinical illness was associated with a rapid expansion of memory B cells (MBCs) in a recall response and the production of anti-parasite IgG1 that was able to mediate clearance of parasitized RBCs. Antibodies were also detected that specifically reacted with uninfected RBCs, consistent with them being a causal factor in the observed anaemia. Specific B-cell subpopulations and immunoglobulin isotypes were identified among the peripheral blood mononuclear cells. As in humans, the B cell subpopulations identified included: naive (IgD+CD27−), unswitched memory (USM: IgD+CD27+), switched memory (SM: IgD−CD27+), and double-negative B cells (DN: IgD−CD27−). The USM and SM B cells dramatically and rapidly expanded during relapses, including IgG+SM B cells, and IgM+SM B cells though fewer in number; in contrast, only IgG+SM B cells increased during the homologous reinfections, however, IgM recognizing both infected and uninfected RBCs was significantly increased. Overall, the reduction of infected RBCs in the peripheral blood by phagocytosis coincided with specific MBC and antibody responses and the lack of clinically detectable illness in both relapses and homologous reinfections. Having generated insights into the B-cell dynamics involved in the generation of strain-specific immune memory, E23 and E24 informed new possible directions to understand B-cell dynamics and functions in future experiments.

Experiment 25 was subsequently performed to test the hypothesis that protective recall responses projected (and then observed) for E24 would not function with a heterologous challenge. The six rhesus monkeys that had been infected in E23 and then re-infected in E24 with the M/B strain of *P. cynomolgi*, were inoculated a few months later with 2,000 freshly isolated *P. cynomolgi* sporozoites of the Ceylon strain; the analysis and integration of the results from this project are in progress, alongside validation experiments (Joyner et al. pers. commun.). For this series of experiments, rectal swabs were also acquired and microbiome and multi-omic integrative analyses are underway to understand host–parasite–microbiome relationships (Cordy et al. pers. commun.).

This series of longitudinal experiments nicely demonstrates the value of this NHP model for exploring immunity to relapsing malaria species, beyond what can typically be done with humans, especially because in the animal model the sporozoite inoculations, treatment of LSFs, and monitoring of blood-stage infections can be rigorously controlled. In a broad sense, these longitudinal macaque infections with *P. cynomolgi* mirror clinical manifestations and immunity results observed in humans with *P. vivax* (reviewed in [25]). In recent years, this NHP model has permitted, for the first time, a close look into the dynamic NAI adaptive responses that curtail clinical exacerbation of disease and pathology, as well as the B cell phenotypes and dynamics that define recall responses responsible for reducing parasitaemia [139]. In future longitudinal *P. cynomolgi* infection experiments, MBC subpopulations and their functionality could be studied in greater depth, including with the sequencing of the B-cell receptors to gain information on their specificity against specific antigens.

Importantly, E23 also capitalized on the opportunity to evaluate the reduction in relapse asexual-stage compared to sexual-stage parasitaemia—assessed by blood-smear counting of gametocytes and transcriptome analysis of gametocyte transcripts [139]. The reduction in parasitaemia during relapses coincided with a reduction in the number of circulating gametocytes, however, the cumulative proportion of gametocytes compared to asexual-stage parasites increased during relapses. These results raise questions as to whether and to what degree relapse infections in humans are clinically silent but infectious by providing a gametocyte reservoir for biting *Anopheles* mosquitoes [139]. It is remarkable—as deduced from the NHP model experiments—that the parasite and host seem to have co-evolved to survive together with non-sterilizing protective host immunity that enables transmission of gametocytes to mosquitoes and thus the propagation of the parasite species. Transmission experiments are now warranted to clarify and understand the combined NHP host–parasite–vector relationships in light of possible intervention strategies to prevent relapsing parasitaemias that can support transmission. Strategically controlled and monitored sequential mosquito feedings (on infected animals or their infected blood, at multiple time points) can confirm levels of infectivity, and, in concert with systems immunological approaches can lead to an improved understanding of how the primed immune system permits relapsing infections and how the parasites sufficiently evade memory recall responses in order to transmit gametocytes to mosquitoes. Future studies can also be envisioned that delve into gametocyte biology and immunity against the sexual stages, providing possible new insights pertinent to understanding transmission reduction or enhancement activities that can be related to *P. vivax* and *P. falciparum* infections (reviewed in [296]).

#### Experiments 06, 07, E30, E33, E35: *Plasmodium knowlesi* infection of *M. mulatta* and *M. fascicularis*

Peterson et al. delved into the pathogenesis of *P. knowlesi* infections that had been initiated with Malayan strain sporozoites in *M. fascicularis* of Mauritius origin and demonstrated that these natural macaque hosts developed clinical illness [268]. However, the animals recovered without anti-malarial intervention. One of the most striking findings was that these animals—unlike *M. mulatta* [277]—did not display evidence of dyserythropoiesis, and in fact, their bone marrow responded when erythropoietin levels were elevated. This response is in direct contrast to what occurs in rhesus macaques infected with *P. coatneyi* or *P. cynomolgi* [130, 131, 139, 140]. One can envision future comparative systems biological studies assessing multiple macaque hosts with the goal of understanding the molecular networks that are affected and the factors that contribute to bone marrow disruption. Indeed, comparisons of peripheral blood transcriptional responses from *P. knowlesi* infected *M. mulatta* and *M. fascicularis*, analysed temporally, show dramatic differences at the onset of the acute infection that seem to support the recovery of *M. fascicularis*, whereas *M. mulatta* continues to show inflammatory and anti-inflammatory processes [396]. Perhaps these distinct responses reflect in part the differences observed in the bone marrow. Furthermore, *M. fascicularis* responded to the parasite as early as 3 days after inoculation with sporozoites, unlike *M. mulatta*, showing early cytokine signaling along with interferon responses while the parasites were still in the liver. Among other findings, Gupta and colleagues also report pre-infection differences in neutrophils and naïve CD4+T cells that lead to differences in Ca^2+^ homeostasis, which ultimately balances inflammation and cell proliferation during the log phase of parasitaemia expansion [413]. These *P. knowlesi* infection experiments also incorporated unique telemetry measurements generated from implanted devices that continuously tracked temperature, blood pressure, heart rate and activity levels (Brady et al. pers. commun.).

#### Comparisons across experiments based on different NHP host–parasite models

The MaHPIC established that the development of anaemia in rhesus macaques infected with *P. coatneyi*, *P. cynomolgi,* or *P. knowlesi* replicates the hemoglobin kinetics of humans with *P. falciparum* or *P. vivax* [130, 131, 139, 140, 277]. During the acute phase of infection, rhesus macaques show evidence of inefficient erythropoiesis, which is further complicated by the removal of uninfected RBCs. Interestingly, the most severe phase of anaemia sets in when erythropoiesis is restored, implying that removal of uninfected RBCs may be the largest contributor to malarial anaemia, as hypothesized in 1999 [44]. Indeed, mathematical modelling of longitudinal infection data by the MaHPIC has supported this hypothesis [112, 489, 494, 495]. These findings suggest that searches are warranted for an adjunctive therapy for severe malarial anaemia that would prevent the removal of uninfected RBCs. Further systems-based studies detailing in vivo mechanisms that cause the removal of uninfected RBCs could be informative, particularly with the advancement of single-cell technologies that allow the fluorescent labelling of erythrocytes with the goal of isolating the phagocytes that take them up in different tissues. In addition, proteomic and other experiments could be applied to identify possible targets on the uninfected RBC surfaces that could be blocked by future antibody-based therapies to prevent their removal by phagocytes.

Data from E03 and E04 were also studied together to determine what common changes may occur in the host due to *Plasmodium* infection regardless of the infecting species. In one example, Tang et al. interpreted peripheral blood transcriptomics data from E03 and E04 by use of a dynamic model of purine metabolism and showed how gene expression associated with purine metabolism from each of these experiments was reflected in specific downstream alterations in metabolomic signatures [496]. Specifically, a main finding was a pattern of flux rearrangement within the purine pathway system that increased production and excretion of inosine, hypoxanthine, and xanthine. More pronounced changes in the flux patterns were associated with higher parasitaemias and the possible relevance of changes in purine metabolism with regards to inflammation and parasite proliferation was discussed. A similar analysis involving human data showed consistent trends in the flux patterns [496]. Other examples show how transcriptomics and metabolomics data from MaHPIC’s NHP experiments are reflected in data from human studies [131, 483].

## Conclusions

### The future of malaria immunology and vaccine development—if we can go to the moon…?!

In 2019 many celebrated the 50th anniversary of the first landing of humans on the moon, and another USA-led trip to the moon was projected for 2024. A year later, the SARS-CoV-2/Covid-19 pandemic struck hard and inspired the development, testing, approval, production and distribution of multiple SARS-CoV-2/Covid-19 vaccines in a matter of months [497] and such research continues with intensity to prevent future pandemics [498]. If scientists and supporting professionals can achieve such ‘unrealistic’ feats during a deadly pandemic, why can’t they make and deliver effective malaria vaccines that are direly required for the eradication of a disease that kills hundreds of thousands every year? Or can they? Is the eradication of malaria simply a matter of will, persistence, and enough funding? Parasitic infections are arguably more complex than viral infections, but the hope persists that scientists will discover an Achille’s heel(s) of the parasite, which then can be exploited as a vaccine target(s), and possibly for the future development of host-directed therapies that will save millions of lives worldwide and greatly increase the chances for malaria eradication. How such elusive targets might be found is of course unknown, but, no doubt, ample global research funding and resources must be maintained to achieve such goals [499–501] (see Box 2).

**Table:** 

Box 2 Millennials, generation Z and beyond!
The future will soon rely on Millennials (born between 1981 and 1996) and Generation Z (born from 1997 onward) [502]. Is it possible today to speed up research so that the next several decades, led by these new generations of scientists, yield exponential growth in understanding the full, multi-faceted extent of malaria? Can we envision an equivalent of ‘going to the moon’ within this time frame? If so, what will that take? Modern methods of systems immunology and vaccinology hold great promise, if they are supported by computational modelling capabilities that allow efficient analysis, integration, and interpretation of large diverse datasets, and offer the hope of revealing insights not possible otherwise. For sure, the field needs patience, time, and resolve, to realize its potential. Funding bodies, as well as passionate wealthy individuals and philanthropically inclined companies, need to recognize and embrace the prospect of a long haul, combined with an innovative, risk-taking mindset of ‘tackling this challenge,’ ‘doing things differently,’ and ‘not being deterred.’ Such research—including studies involving NHPs—has so far advanced in fits and starts. Funding comes, and funding goes. Capable dedicated research teams form, specialized training occurs, projects gain momentum, and then agendas veer off. This constant ebb and flow of research funding makes it difficult to maintain strong active research teams. If the moon-shot goal of malaria research is disease eradication, the future of today’s post-genomics era will require an expansion of research capabilities and talents, with individuals and teams able to analyse and decipher meaning from the vast amounts of genomic, epigenomic, and other data types that can be generated, for instance, in the course of longitudinal infections. To advance malaria research with the use of NHPs, NHP and malariology expertise must be sustained and nurtured, and this expertise must be augmented with a deepened knowledge of the concepts and approaches of immunology, vaccinology, computational biology, and systems biology. The training and cultivation of junior scientists as capable leaders in this research area is essential—now. This sentiment was expressed over 12 years ago by the CDC’s preeminent malaria parasite NHP infection expert and entomologist William Collins [503], a co-author of “The Primate Malarias”. The same is now expressed by this author, as she nears retirement!

Malaria research interests and agendas have become all-encompassing with consideration of today’s malaria eradication goals. The pursuit of effective vaccines continues alongside epidemiological tracking of parasites, species and infections, and focused attention on the need to detect and treat relapse and chronic asymptomatic cases of malaria. Improved diagnostics, drugs and issues relating to insecticide availability and resistance are also at the forefront. New efforts include rapid species-specific detection of infections using non-invasive procedures [504, 505], the identification of biomarkers of immunity and disease [51, 131, 506–509], and genetically altered mosquitoes that do not transmit the disease [270]. The need continues for new drugs and drug combination therapies in the face of drug resistance, or contraindications, as with primaquine or tafenoquine use in pregnant and G6PD deficient individuals [10], as well as insecticide-treated nets, other vector inhibitory tools, and procedures for the monitoring and evaluation of epidemiological studies and clinical trials. In addition, the nationwide and regional epidemiological, ecological, socioeconomic, and cultural factors must be considered in the 100 or so countries with malaria.

As a result of the recent SARS-CoV-2/Covid-19 pandemic, communities and their malaria control/elimination/eradication agendas have been disrupted in countless unexpected ways [271] and many questions have been raised on evaluating, managing, and treating syndemics such as the one between malaria and SARS-CoV-2/Covid-19 [272]. With signs that the SARS-CoV-2/Covid-19 pandemic is coming under control, it is time to reassess research directions to keep advancing the goals of malaria eradication. As has been learned with the SARS-CoV-2/Covid-19 pandemic experience, it is wise to stay ahead of predictable epidemics – and pandemics. Scientific and public health experts must keep working together, mastering and advancing key knowledge about malaria and devising ways forward to prevent, treat, and eliminate the disease.

Today with the benefits of cellular imaging, -omic technologies, and computational methods, scientists can visualize biological functions and dynamics, in essence beginning to gain traction on understanding complex in vivo dynamical systems. The advent of systems biological approaches offers hope that it is possible to capitalize on NHP model systems hand-in-hand with CHMI studies. Notwithstanding, despite advances recognized in this review, the use of NHPs for malaria research has arguably been quite limited to date, especially compared to other infectious diseases of widespread importance. Any reluctance is unfortunate because clear evidence is mounting that NHPs can be an effective tool to tease out the details of NAI, VCI and pathogenesis in controlled longitudinal infection studies. As mechanistic depth is required to address many hypotheses regarding the molecular basis of host–parasite interactions, necessitating rigorous sampling of blood, bone marrow, or other tissues, NHPs may be the preferred host of the future.

It is especially timely now for more in-depth studies to better understand NAI to *P. vivax* primary infections, relapses, and repeat infections using robust Old World macaque-*P. cynomolgi* infection models [168, 190], along with or followed by confirmatory/validation experiments with *P. vivax* in the New World monkeys [20]. Likewise, Old World macaque-*P. coatneyi* and -*P. knowlesi* infection models are excellent for studying acute and chronic immune responses and pathogenesis, as well as antigenic variation based on the large Schizont Infected Cell Agglutination variant antigens (*SICAvar*) multigene family that corresponds to the *var* gene family in *P. falciparum* [92, 94]. Malaria during pregnancy has been studied using *P. coatneyi*, *P. cynomolgi* and *P. knowlesi* in rhesus monkeys and *P. knowlesi* in baboons (reviewed in [273]), and future studies on immunity and pathogenesis would also be of value with these models.

Comparative studies with different parasite species, strains, and co-infections, possibly involving animals of different age groups and sexes (infants, juveniles, adults), could also be fruitful toward understanding NAI and VCI in these populations. Such studies may also lead to insights supporting the development of vaccines that provide species- or strain-transcending immunity, comparable to multiple natural exposures in malaria endemic areas to homologous or heterologous parasites that can result in the building of NAI, with a lack of symptomology. While this level of immunity would be a major achievement toward eradication, the problem of asymptomatic infections and chronicity must also be addressed. Otherwise, transmission will remain a likely fact, and the chances for eradication will be much reduced. Interventions focused on NAI goals must include both anti-disease and anti-parasite immunity, as well as the elimination of infectious gametocytes.

A caveat for studying the cellular dynamics within the immune system while relying solely on peripheral blood or bone marrow is that not all immune cell types of interest, or their specific activated phenotypes, are necessarily circulating in the blood or present in the bone marrow. Moreover, a subset of iRBCs can become sequestered or concealed in various tissues and organs [24, 112, 151, 152, 267, 268, 274, 277], so that peripheral blood parasitaemia counts do not necessarily reflect the true parasite load in the host (reviewed in [24, 97, 110, 196]). Tissue samples other than peripheral blood can seldom be obtained from humans. However, various tissues can be accessed from infected NHPs with biopsy procedures or during necropsies in terminal studies. Future experiments are warranted to assess the different cell type niches for *Plasmodium* and their functional attributes in NHP infections. It will be informative to assess these at several time points during infections, when possible, from sequential biopsies on individual animals, or from a cohort of animals with staggered terminal analyses, such that the cell types within different tissues could be examined. These studies can be performed, for example, during the liver stage and acute and chronic stages of infections, post treatment, and during relapses in the case of *P. vivax* or *P. cynomolgi*. With such analyses, a holistic picture of the dynamics of infection, immunity and disease will take shape. Similarly, future iterations of the types of experiments carried out by the MaHPIC will permit the pursuit of hypothesis-driven questions that will gradually become more specific. Thus, within the limits imposed by ethical regulations, more information will be gained from a specific period of an infection or from a specific tissue or cell type and targeted studies will yield insights into the living tissue architecture and the parasite’s environment.

The ever-elusive efficacious malaria vaccine, which can be counted on for complete protection against one or all species, strains and variants of *Plasmodium*, may not be in the realm of realistic possibilities—today. However, with greater fundamental understanding of the workings of the immune system and host–parasite interactions, strategic, fortuitous—or serendipitous—discoveries will pave the way forward in ways unbeknown today. All things considered, from the hundreds of millions of *Plasmodium* infections and clinical cases of malaria that still occur annually in close to 100 countries [1], the goals of regional elimination and global eradication [160], and the potential today for incrementally deep exploration into host–pathogen interactions and the identification of new intervention targets and strategies (parasite- or host-directed), it is important to capitalize on the value of multiple animal models for bringing critical knowledge to the fight against malaria. In this vein, Zuck and colleagues boldly concluded that “the study of host–pathogen interactions presents an unmatched opportunity for the field of systems biology, just as the approach of systems biology presents an unmatched opportunity for the eradication of malaria [275].”

Fifty years ago, the authors of “The Primate Malarias” demonstrated much curiosity and dedication, thereby consciously or not paving the way for decades of malaria research involving NHPs to follow their lead. Once again, the malaria research field is at an important juncture, and the question is whether society has the determination to utilize all available resources and the cornucopia of amazing new technologies to advance our understanding of malaria, immunity, and pathogenesis for the benefit of translational research that will ultimately benefit communities affected by this disease worldwide. The catch phrase “use them or lose them” needs to be taken to heart when it comes to NHP model systems. The discussion of advances based on NHP models described here is intended to inspire curiosity and the pursuit of new avenues toward knowledge regarding the multi-faceted system that encompasses malaria. The necessary tools are emerging. They are powerful and unprecedented, but they will only be efficacious if they are used and relentlessly refined and improved by new cohorts of well-trained malariologists. This article’s goal—while honoring leaders from the past—is thus a call to action. The longitudinal infection experiments performed by the MaHPIC are an example of the benefits reaped from decades of prior research with NHPs. It was timely in 2012 for the MaHPIC team to begin developing the systems biological underpinnings for longitudinal NHP infection research, and their work added new dimensions of understanding to key aspects of the disease, such as relapses, chronic infections, anaemia, and immune memory. However, much is yet to be learned, venturing into uncharted territory. If technologies continue to advance as expected, and the tenacity for new research using NHPIMs remains strong, insights into malaria in these models will continue to provide a multi-dimensional holistic view of parasitism at the cellular, tissue and organ levels, and spawn a wider, and at the same time sharper focus onto possible targets for interventions and disease eradication. Terabytes of data from MaHPIC experiments have been deposited in the NIAID-supported Bioinformatics Resource Centers, PlasmoDB [420, 276] and other public databases for the benefit of the global research community. Many opportunities now exist for the analysis and re-analysis of data across MaHPIC’s clinical and NHP experiments [482].

## Data Availability

Not applicable.

## Abbreviations

atMBC: Atypical memory B cells
CDC: Centers for Disease Control and Prevention
CHMI: Controlled human malaria infection
CSP: Circumsporozoite protein
iRBC: Infected red blood cell
MaHPIC: Malaria Host–Pathogen Interaction Center
MBC: Memory B cell
NAI: Naturally acquired immunity
NHP: Nonhuman primate
NHPIM: Nonhuman primate infection model
NPRC: National Primate Research Center
PBMCs: Peripheral blood mononuclear cells
RBC: Red blood cell
SICA: Schizont-infected agglutination antigen
*SICAvar*: *Schizont-infected agglutination variant antigen gene*
VCI: Vaccine candidate-induced immunity
*var*: *Variant antigen gene*
YNPRC: Yerkes National Primate Research Center
